# Reprogramming of the FOXA1 cistrome in treatment-emergent neuroendocrine prostate cancer

**DOI:** 10.1038/s41467-021-22139-7

**Published:** 2021-03-30

**Authors:** Sylvan C. Baca, David Y. Takeda, Ji-Heui Seo, Justin Hwang, Sheng Yu Ku, Rand Arafeh, Taylor Arnoff, Supreet Agarwal, Connor Bell, Edward O’Connor, Xintao Qiu, Sarah Abou Alaiwi, Rosario I. Corona, Marcos A. S. Fonseca, Claudia Giambartolomei, Paloma Cejas, Klothilda Lim, Monica He, Anjali Sheahan, Amin Nassar, Jacob E. Berchuck, Lisha Brown, Holly M. Nguyen, Ilsa M. Coleman, Arja Kaipainen, Navonil De Sarkar, Peter S. Nelson, Colm Morrissey, Keegan Korthauer, Mark M. Pomerantz, Leigh Ellis, Bogdan Pasaniuc, Kate Lawrenson, Kathleen Kelly, Amina Zoubeidi, William C. Hahn, Himisha Beltran, Henry W. Long, Myles Brown, Eva Corey, Matthew L. Freedman

**Affiliations:** 1 Department of Medical Oncology, Dana-Farber Cancer Institute, Boston, MA USA; 2 The Eli and Edythe L. Broad Institute, Cambridge, MA USA; 3 Center for Functional Cancer Epigenetics, Dana-Farber Cancer Institute, Boston, MA USA; 4 Laboratory of Genitourinary Cancer Pathogenesis, Center for Cancer Research, National Cancer Institute, NIH, Bethesda, MD USA; 5 Department of Obstetrics and Gynecology and the Women’s Cancer Program at the Samuel Oschin Comprehensive Cancer Institute, Cedars-Sinai Medical Center, Los Angeles, CA USA; 6 Center for Bioinformatics and Functional Genomics, Department of Biomedical Sciences, Cedars-Sinai Medical Center, Los Angeles, CA USA; 7 Department of Pathology and Laboratory Medicine, David Geffen School of Medicine, University of California Los Angeles, Los Angeles, CA USA; 8 Istituto Italiano di Tecnologia, Genova, Italy; 9 Department of Oncologic Pathology, Dana-Farber Cancer Institute, Boston, MA USA; 10 Department of Urology, University of Washington, Seattle, WA USA; 11 Divisions of Human Biology and Clinical Research, Fred Hutchinson Cancer Research Center, Seattle, WA USA; 12 Department of Data Sciences, Dana-Farber Cancer Institute, Boston, MA USA; 13 Department of Biostatistics, Harvard T.H. Chan School of Public Health, Boston, MA USA; 14 Department of Pathology, Brigham & Women’s Hospital and Harvard Medical School, Boston, MA USA; 15 Vancouver Prostate Centre, Vancouver, BC Canada; 16 Department of Urologic Sciences, Faculty of Medicine, University of British Columbia, Vancouver, BC Canada

**Keywords:** Prostate cancer, Epigenomics

## Abstract

Lineage plasticity, the ability of a cell to alter its identity, is an increasingly common mechanism of adaptive resistance to targeted therapy in cancer. An archetypal example is the development of neuroendocrine prostate cancer (NEPC) after treatment of prostate adenocarcinoma (PRAD) with inhibitors of androgen signaling. NEPC is an aggressive variant of prostate cancer that aberrantly expresses genes characteristic of neuroendocrine (NE) tissues and no longer depends on androgens. Here, we investigate the epigenomic basis of this resistance mechanism by profiling histone modifications in NEPC and PRAD patient-derived xenografts (PDXs) using chromatin immunoprecipitation and sequencing (ChIP-seq). We identify a vast network of *cis*-regulatory elements (*N*~15,000) that are recurrently activated in NEPC. The FOXA1 transcription factor (TF), which pioneers androgen receptor (AR) chromatin binding in the prostate epithelium, is reprogrammed to NE-specific regulatory elements in NEPC. Despite loss of dependence upon AR, NEPC maintains FOXA1 expression and requires FOXA1 for proliferation and expression of NE lineage-defining genes. Ectopic expression of the NE lineage TFs ASCL1 and NKX2-1 in PRAD cells reprograms FOXA1 to bind to NE regulatory elements and induces enhancer activity as evidenced by histone modifications at these sites. Our data establish the importance of FOXA1 in NEPC and provide a principled approach to identifying cancer dependencies through epigenomic profiling.

## Abstract (web summary)

The molecular processes that lead to neuroendocrine prostate cancer after treating prostate adenocarcinoma (PRAD) are not well understood. Here the authors show that regulation by FOXA1 and changes in the epigenomic profile drive the transition from PRAD to a neuroendocrine phenotype.

## Introduction

In recent years, potent AR pathway inhibitors have extended the survival of patients with metastatic prostate cancer^1,2^. Prostate tumors inevitably escape AR inhibition through reactivation of AR signaling or, increasingly, via lineage plasticity^3,4^. The mechanisms underlying lineage plasticity remain unclear but likely involve transdifferentiation of PRAD to NEPC rather than de novo emergence of NEPC. NEPC and PRAD tumors from an individual patient share many somatic DNA alterations, implying a common ancestral tumor clone^5^. While the genomic profiles of NEPC and PRAD are relatively similar, their gene expression profiles and clinical behavior differ markedly^6^. We therefore set out to characterize epigenomic differences between NEPC and PRAD, hypothesizing that reprogramming of distinct regulatory elements drives their divergent phenotypes.

In this study, we profile histone modifications in NEPC and PRAD patient-derived xenografts (PDXs), identifying ~15,000 regulatory elements that are dormant in PRAD but consistently activated in NEPC. A significant portion of NEPC-enriched regulatory elements are bound in NEPC by FOXA1, a transcription factor associated with prostate development and AR-mediated transcription^7,8^. The FOXA1 cistrome, or set of binding sites, is extensively reprogrammed in NEPC, with loss of FOXA1 binding at PRAD-enriched regulatory elements and gain of FOXA1 at NEPC-enriched regulatory elements. Unexpectedly, FOXA1 remains active in NEPC despite the loss of luminal identity and AR expression. FOXA1 is necessary for proliferation and neuroendocrine gene expression in experimental models of NEPC. Ectopic expression of NEPC-associated transcription factors is sufficient to reprogram the FOXA1 cistrome in PRAD to resemble its counterpart in NEPC and activate NEPC transcriptional programs. Our data indicate a dependency of NEPC upon FOXA1, which may have therapeutic implications.

## Results

### Comparative epigenomic profiles of PRAD and NEPC

We performed ChIP-seq for the histone post-translational modification H3K27ac to identify active regulatory elements in the LuCaP PDX series^9^, a set of xenografts derived from advanced PRAD (*N* = 22) and treatment-emergent NEPC (*N* = 5). We identified a median of 55,095 H3K27ac peaks per sample (range 37,599–74,640) (Supplementary Data 1). Notably, the transcriptomes of the LuCaP PDXs reflect differences in gene expression observed between clinical PRAD and NEPC metastases (Supplementary Fig. 1a), indicating their relevance to clinical prostate cancer.

**Fig. 1:**
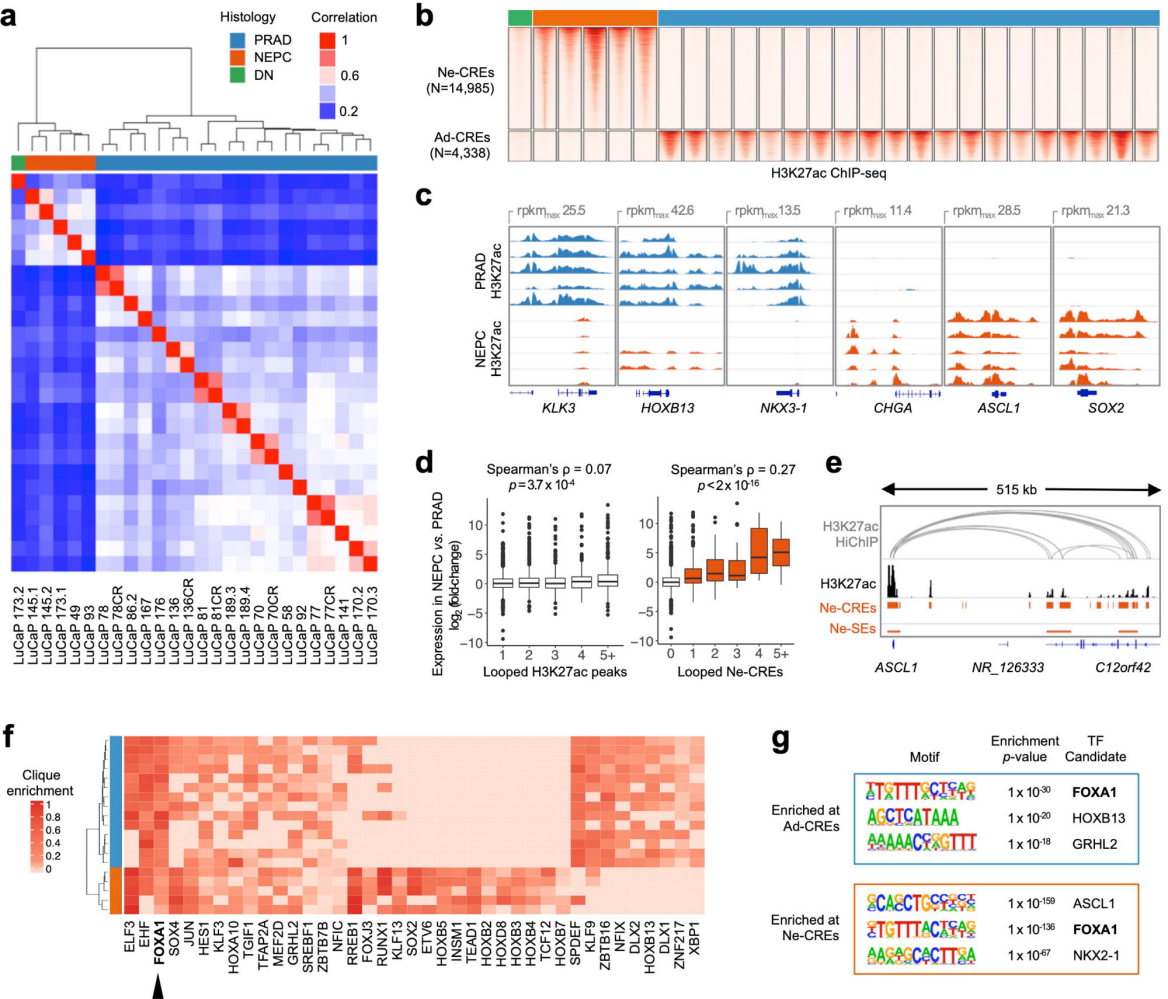
Epigenomic divergence of PRAD and NEPC. **a** Hierarchical clustering of PRAD and NEPC based on sample-to-sample correlation of H3K27ac profiles. “DN” (“double-negative”) indicates a LuCaP PDX without AR or NE marker expression (see also Supplementary Fig. 1). **b** Heatmaps of normalized H3K27ac tag densities at differentially H3K27-acetylated regions (±2 kb from peak center) between NEPC and PRAD. “CREs” signify candidate regulatory elements. **c** H3K27ac signal near selected prostate-lineage and NEPC genes. Five representative samples from each histology are shown. **d** Differential expression (NEPC vs. PRAD) of genes with the indicated number of distinct looped H3K27ac peaks (left) or Ne-CREs (right) detected by H3K27ac HiChIP in LuCaP 173.1 (NEPC). Box boundaries correspond to 1st and 3rd quartiles; whiskers extend to a maximum of 1.5x the inter-quartile range. Two-sided Wilcoxon *p*-value is indicated for comparison of genes with loops to one Ne-CRE or H3K27ac peak versus two or more. **e** H3K27ac HiChIP loops in LuCaP 173.1 from *ASCL1* to Ne-CREs and NEPC-restricted super-enhancers (Ne-SEs). H3K27ac tag density for LuCaP 173.1 is shown in black. **f** Candidate master transcription factors in NEPC and PRAD based on regulatory clique enrichment (see methods). **g** Three most significantly enriched nucleotide motifs present in >10% of Ad-CREs or Ne-CREs by de novo motif analysis. Source data are provided as a Source Data file.

Unsupervised hierarchical clustering and principal component analysis based on genome-wide H3K27 acetylation cleanly partitioned NEPC and PRAD LuCaP PDXs (Fig. 1a and Supplementary Fig. 1b, c). We identified 14,985 sites with eight-fold or greater increases in H3K27 acetylation in NEPC compared to PRAD at an adjusted *p*-value of 10^−3^. We termed these sites neuroendocrine-enriched candidate regulatory elements (“Ne-CREs”; Fig. 1b, Supplementary Data 2, and Supplementary Fig. 1d). A smaller set of sites (4338) bore greater H3K27ac signal in PRAD (termed “Ad-CREs”). Liver metastases from clinical NEPC and PRAD demonstrated enrichment of H3K27ac at Ne-CREs and Ad-CREs, respectively, confirming that the LuCaP PDX models reflect lineage-specific epigenomic features of clinical prostate tumors (Supplementary Fig. 1e).

Ad-CREs were found near prostate-lineage genes such as *KLK3*, *HOXB13*, and *NKX3-1*, while Ne-CREs resided near genes enriched for neuronal and developmental annotations, including *CHGA*, *ASCL1*, and *SOX2*^10^ (Fig. 1c and Supplementary Table 1). Genes with higher expression in NEPC compared to PRAD were enriched for nearby Ne-CREs (Supplementary Fig. 1f) and formed three-dimensional contacts with a greater number of Ne-CREs as assessed by H3K27ac HiChIP (Fig. 1d, Supplementary Fig. 1g, h, and Supplementary Data 3 and 4). For example, *ASCL1*, which encodes a neural lineage TF that is highly upregulated in NEPC (Supplementary Fig. 1a), interacts with 15 gene-distal Ne-CREs between 280 and 465 kb telomeric to *ASCL1*, including two NEPC-restricted super-enhancers within intronic regions of *C12ORF42* (Fig. 1e). These results suggest that Ne-CREs regulate neuroendocrine transcriptional programs through interaction with NEPC gene promoters.

We nominated candidate TFs that may orchestrate NEPC lineage gene expression by binding to Ne-CREs. Lineage-defining TF genes often reside within densely H3K27-acetylated super-enhancers^11^ and form core regulatory circuits, or “cliques”, by mutual binding of one another’s *cis*-regulatory regions^12,13^. Several TFs showed clique enrichment specifically in NEPC (Fig. 1f) and/or were encompassed by NEPC-restricted super-enhancers (Supplementary Fig. 2), including known NE lineage TFs (e.g., *ASCL1* and *INSM1*) and candidates such as *HOXB2-5*.

### FOXA1 is an essential transcription factor in NEPC

**Fig. 2:**
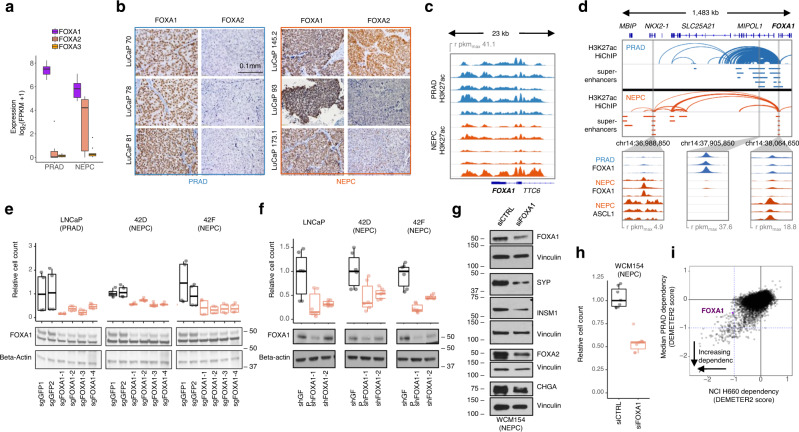
FOXA1 remains a critical lineage transcription factor in NEPC. **a** Transcript expression of FOXA family TFs in LuCaPs PDXs (five NEPC and five PRAD; two replicates each). **b** FOXA1/FOXA2 immunohistochemistry in six representative PDXs. **c** H3K27ac profiles at *FOXA1* in five representative PRAD and NEPC PDXs. **d** H3K27ac HiChIP loops near *FOXA1* in LuCaP 173.1 (NEPC) and LNCaP (PRAD). Bars indicate super-enhancers in five representative LuCaPs of each lineage. Blowups show ChIP-seq read pileups for FOXA1 and ASCL1 in PDXs of the indicated lineage. **e**, **f** Proliferation of LNCaP and 42D/42F derivatives with inactivation of FOXA1 by CRISPR (**e**) or shRNA (**f**) across two independent experiments (*n* = 6 replicates). Numbers next to western blots indicate molecular weight markers (kD). **g**, **h** Proliferation (**g**) and expression of neuroendocrine marker proteins (**h**) with siRNA knock-down of FOXA1 in the NEPC organoid model WCM154. Knock-down was repeated in two independent experiments with similar results. **i** Essentiality of genes in NCI-H660 (NEPC) versus PRAD cell lines in a published shRNA screening dataset^72^. More negative DEMETER2 scores indicate greater dependency. The blue lines indicate the median DEMETER2 score for pan-essential genes. For all boxplots, box boundaries correspond to 1st and 3rd quartiles; whiskers extend to a maximum of 1.5x the inter-quartile range. Source data are provided as a Source Data file.

Notably, a single TF gene, *FOXA1*, demonstrated clique enrichment in all NEPC and PRAD LuCaP PDXs (Fig. 1f). FOXA1 is a pioneer TF of endodermal tissues^7^ with a critical role in prostate development^8^ but no characterized function in NEPC. The forkhead motif recognized by FOXA1 was the second most significantly enriched nucleotide sequence within Ne-CREs (Fig. 1g). FOXA2, a previously-reported NEPC TF^14^, does not wholly account for the forkhead motif enrichment because FOXA2 was not expressed in several NEPC samples (Fig. 2a, b and Supplementary Fig. 3a). In contrast, FOXA1 was expressed in all NEPCs (Fig. 2a, b and Supplementary Table 2), as well as in resident neuroendocrine cells of benign prostate tissue (Supplementary Fig. 3).

Multiple lines of investigation supported a pivotal role of FOXA1 in NEPC. A super-enhancer encompassed *FOXA1* in all NEPC LuCaP PDXs (Fig. 2c and Supplementary Fig. 2). In NEPC, the *FOXA1* promoter shed contacts with its regulatory region identified in PRAD^15^ and looped to a distinct NEPC-restricted super-enhancer (Fig. 2d). Both the distal super-enhancer and promoter were co-bound by FOXA1 and ASCL1, suggesting an auto-regulatory circuit that is characteristic of master transcriptional regulators^16^. Suppression of FOXA1 in a variety of NEPC cellular models^17,18^ demonstrated that FOXA1 is essential for cellular proliferation and expression of NE markers, including NE lineage TFs such as FOXA2 and INSM1 (Fig. 2e–h). Analysis of a published shRNA screen confirmed a dependency on FOXA1 in the NEPC cell line NCI-H660 (Fig. 2i). Thus, FOXA1 exhibits several features of a master transcriptional regulator in NEPC.

**Fig. 3:**
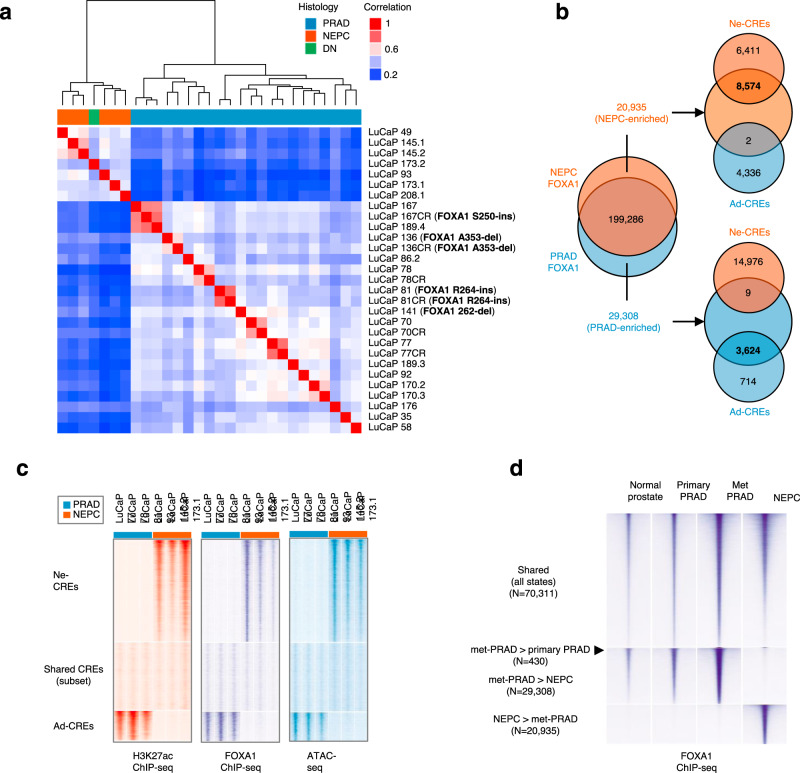
Reprogramming of the FOXA1 cistrome in NEPC. **a** Hierarchical clustering of LuCaP PDXs by FOXA1-binding profiles. “DN” (“double-negative”) indicates a PDX without AR or NE marker expression. FOXA1 mutational status is noted; see also Supplementary Table 3). **b** Venn diagram of lineage-enriched and shared FOXA1-binding sites and their overlap with lineage-enriched candidate regulatory elements (Ad-CREs and Ne-CREs). Differential FOXA1 peaks were identified from *n* = 5 NEPC and *n* = 11 PRAD PDXs. **c** Normalized tag densities for H3K27ac/FOXA1 ChIP-seq and ATAC-seq at Ne-CREs and Ad-CREs. Three representative NEPC and PRAD PDXs are shown. **d** Average normalized tag densities for FOXA1 in normal prostate, primary PRAD, and PDXs derived from PRAD metastases (Met PRAD) or NEPC (five samples in each category) at differential FOXA1-binding sites between these groups. There are insufficient differential sites to display (<100) for the Primary PRAD > Met PRAD comparison and the Primary PRAD vs. Normal prostate comparisons.

We profiled FOXA1-binding sites in NEPC and PRAD using ChIP-seq. FOXA1 relocates to a distinct set of binding sites in NEPC PDXs (Fig. 3a), which overlap with the majority of Ne-CREs (Fig. 3b). In PRAD, Ne-CREs were devoid of FOXA1 binding and heterochromatic as assayed by ATAC-seq, but they acquired FOXA1 binding and chromatin accessibility in NEPC (Fig. 3c). Conversely, Ad-CREs lost FOXA1 binding in NEPC and became less accessible by ATAC-seq. These changes were not due to *FOXA1* mutations, which can promote neuroendocrine gene transcription^19^, because all NEPC LuCaPs contained wild-type *FOXA1* sequences. To contextualize the extent of FOXA1 reprogramming in NEPC, we compared FOXA1-binding profiles in normal prostate epithelium, localized PRAD, and PDXs derived from metastatic PRAD. At the same level of stringency, fewer than 500 sites exhibited differential FOXA1 binding between these categories; by comparison, FOXA1 binding was gained at 20,935 and lost at 29,308 sites in NEPC compared to metastatic PRAD (Fig. 3d).

### NEPC genes contain bivalent promoter marks in PRAD

**Fig. 4:**
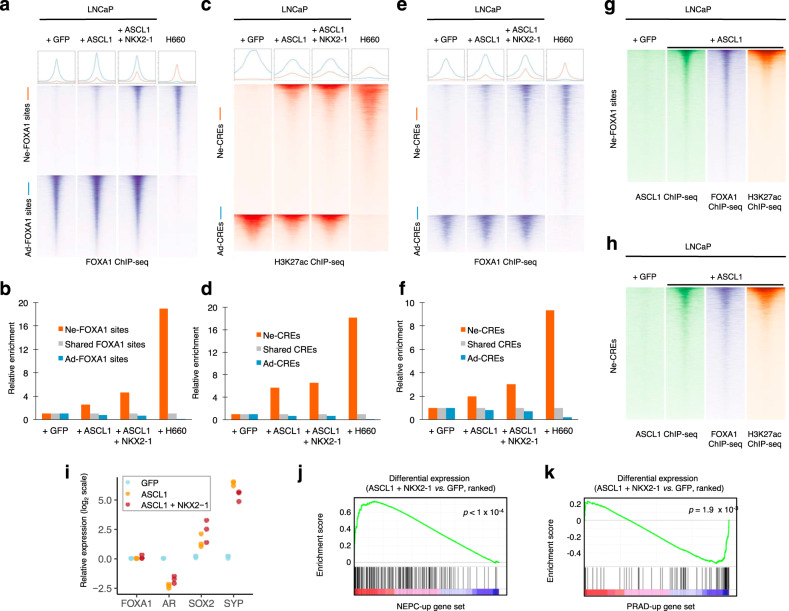
FOXA1 is extensively redistributed at lineage-specific regulatory elements. **a** Normalized ChIP-seq tag density for FOXA1 at NEPC-enriched and PRAD-enriched FOXA1-binding sites under the indicated conditions. Profile plots (top) represent mean tag density at sites depicted in the heatmaps. **b** Enrichment of FOXA1 peaks for overlap with NEPC-enriched and PRAD-enriched FOXA1-binding sites in the indicated conditions, normalized to FOXA1 peaks shared between PRAD and NEPC. **c**–**f** Normalized ChIP-seq tag density for H3K27ac (**c**) and FOXA1 (**e**) at Ne-CREs and Ad-CREs under the indicated experimental conditions. Enrichment of overlap of H3K27ac peaks (**d**) and FOXA1 peaks (**f**) with Ne-CREs and Ad-CREs under the indicated conditions. **g**–**h** Normalized ChIP-seq tag density for ASCL1, FOXA1, and H3K27ac under the indicate experimental conditions at NEPC-enriched FOXA1 sites (**g**) and Ne-CREs (**h**). **i** Effect of ASCL1 overexpression on transcript levels of indicated genes, measured by qPCR. Fold-change relative to +GFP condition is shown, using normalization to GAPDH. Three biological replicates are shown for each condition. **j**–**k** Gene set enrichment analysis of genes upregulated at least 8-fold in LuCaP NEPC (**j**) or PRAD (**k**) at adjusted *p*-value < 10^−18^. Genes are ranked by differential expression between LNCaP + ASCL1 + NKX2-1 and +GFP conditions based on RNA-seq. Unadjusted permutation-based one-sided *p*-values for enrichment are shown. Source data are provided as a Source Data file.

We sought to understand the mechanism by which FOXA1 binding is reprogrammed in NEPC. In addition to DNA sequence, cooperative binding with partner TFs is an important determinant of pioneer factor localization^20^. Since the motifs recognized by ASCL1 and NKX2-1 were highly enriched at Ne-CREs (Fig. 1g), we tested whether overexpression of these TFs in the PRAD cell line LNCaP could induce FOXA1 binding at Ne-CREs. Overexpression of ASCL1 and NKX2-1 (A + N) increased FOXA1 binding at NEPC-enriched FOXA1-binding sites (Fig. 4a, b) and induced H3K27 acetylation of Ne-CREs (Fig. 4c–f). ASCL1 co-localized with FOXA1 at NEPC-enriched FOXA1-binding sites and Ne-CREs (Fig. 4g–h). A+N expression recapitulated global transcriptional changes between NEPC and PRAD, including suppression of *AR* and induction of *SYP* and *CHGA* (Fig. 4i–k). Thus, ectopic expression of ASCL1 and NKX2-1 is sufficient to partially reprogram FOXA1 binding in PRAD to Ne-CREs and induce de novo H3K27 acetylation at these regions, with resultant NEPC gene expression.

**Fig. 5:**
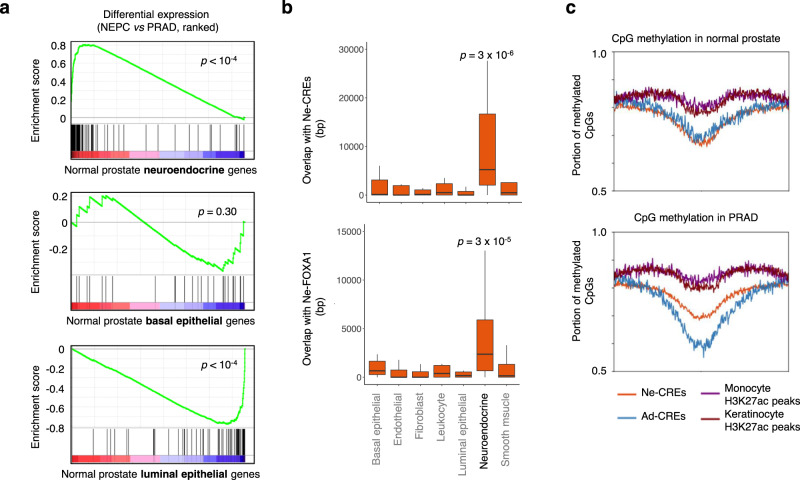
Gene expression of benign prostate cells compared to NEPC transcriptomes and epigenomes. **a** Gene set enrichment analysis of genes specifically expressed in neuroendocrine, basal, and luminal cells from normal prostate^73^. Genes are ranked by differential expression in NEPC and PRAD LuCaP PDXs. **b** Overlap of NEPC-enriched H3K27ac peaks (Ne-CREs; *n* = 14,985; top) and FOXA1-binding sites (Ne-FOXA1; *n* = 20,935; bottom) with a 200 kb windows centered on the transcriptional start sites of the 20 most significantly differentially expressed genes in each indicated prostate cell type^73^. Box boundaries correspond to 1st and 3rd quartiles; whiskers extend to a maximum of 1.5x the inter-quartile range. *p*-values correspond to two-sided Wilcoxon test of Ne-CRE/Ne-FOXA1 peak overlap near neuroendocrine cell genes versus all other indicated gene categories. **c** Fraction of CpG methylation detected by whole-genome bisulfite sequencing in normal prostates tissue and PRAD at Ne-CREs and Ad-CREs. Methylation levels at H3K27ac peaks identified in epithelial keratinocytes or in peripheral blood monocytes are included for comparison. *x*-axis corresponds to peak center ±3 kb.

Despite intense interest, it remains unclear why PRAD can adopt a seemingly unrelated lineage to overcome androgen blockade, while most cancers do not dramatically alter their cellular identity throughout treatment. Lineage tracing studies have demonstrated that the epithelial cells that give rise to PRAD share a common developmental progenitor with resident neuroendocrine cells in the prostate^21,22^. In this common progenitor cell, Ne-CREs and their FOXA1-binding sites might be physiologically poised for activation upon commitment to a neuroendocrine lineage. In support of this model, genes that are highly expressed in normal neuroendocrine prostate cells are also highly expressed in NEPC (Fig. 5a), and are enriched for nearby Ne-CREs and NEPC-restricted FOXA1-binding sites (Fig. 5b). Additionally, Ne-CREs are relatively hypomethylated in normal prostate tissue and PRAD despite absence of H3K27 acetylation, a feature of decommissioned enhancers that were active in development (Fig. 5c)^23,24^.

**Fig. 6:**
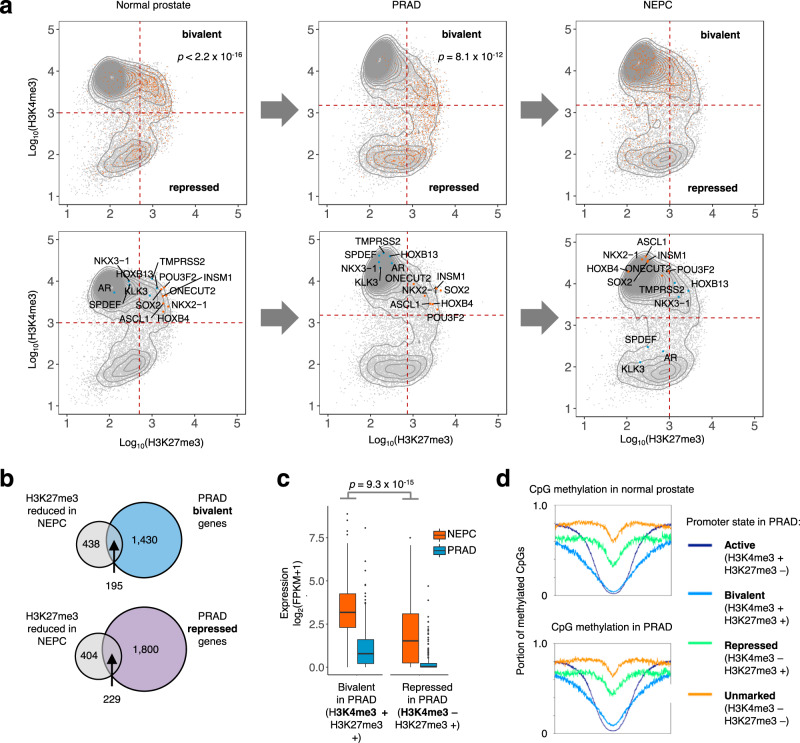
Encoding of neuroendocrine regulatory programs in the developmental history of prostate cancer. **a** Average ChIP-seq tag density in normal prostate (*n* = 3 samples), PRAD (*n* = 5) and NEPC (*n* = 5) for H3K4me3 and H3K27me3 within 2 kb of a gene transcriptional start site (TSS). Each dot represents a unique gene TSS. The top row highlights genes with upregulated expression in NEPC compared to PRAD (orange). *p*-values indicate Pearson’s Chi-squared test comparing enrichment of upregulated genes within the “bivalent” quadrant compared to the bottom two quadrants. Selected genes are highlighted in the bottom row. **b** Intersection of genes with bivalent (H3K27me3^+^/H3K4me3^+^) or repressed (H3K27me3^+^/H3K4me3^−^) promoter annotations in PRAD and genes with reduced promoter H3K27me3 in NEPC vs. PRAD (log_2_ fold-change <−1, FDR-adjusted *p*-value = 0.01). **c** Transcript expression levels in NEPC of genes whose promoters lose H3K27me3 in NEPC compared to PRAD. Genes are grouped by bivalent (*n* = 1625) or repressed (*n* = 2029) promoter annotations in PRAD. Box boundaries correspond to 1st and 3rd quartiles; whiskers extend to a maximum of 1.5x the inter-quartile range. *p*-value corresponds to two-sided Wilcoxon rank-sum test. **d** Fraction of CpG methylation in normal prostate tissue and PRAD at TSS ±3 kb for genes in each indicated category. Source data are provided as a Source Data file.

We hypothesized that a neuroendocrine epigenomic program is encoded in the developmental history of the prostate, thereby priming NEPC genes for inappropriate activation under the selective pressure of androgen blockade. Consistent with this hypothesis, many genes that become highly expressed in NEPC have “bivalent” (H3K4me3^+^/H3K27me3^+^) promoter histone marks in normal prostate tissue and PRAD (Fig. 6a). Bivalent genes are thought to be poised for lineage-specific activation upon removal of H3K27me3 at the appropriate stage of development^25,26^. Our data suggested that a similar principle underlies transcriptional changes in neuroendocrine differentiation of prostate cancer. H3K27me3 levels decreased in NEPC compared to PRAD at 633 gene promoters, which were enriched for binding sites of the REST repressor of neuronal lineage transcription^27^ (Supplementary Fig. 4). Similar numbers of these promoters were bivalent (H3K4me3^+^/H3K27me3^+^; *n* = 195) and repressed (H3K4me3^−^/H3K27me3^+^; *n* = 229) in PRAD (Fig. 6b). Critically, however, genes with bivalent (H3K4me3^+^) promoters in PRAD became more highly expressed in NEPC (Fig. 6c) than H3K4me3^−^ genes. These bivalent genes, which included NEPC TFs *ASCL1, INSM1*, and *SOX2*, may have been prepared for activation in the development of a prostate progenitor cell. Their residual H3K4me3 and promoter hypomethylation (Fig. 6d) suggest heightened potential for reactivation^24^ in NEPC with the disruption of pro-luminal AR-driven transcriptional programs.

## Discussion

In summary, our work demonstrates that the *cis*-regulatory landscape of prostate cancer is extensively reprogrammed in NEPC. Epigenomic profiling of human NEPC xenografts supports a central role of FOXA1 in this reprogramming. The FOXA1 cistrome shifts dramatically between NEPC and PRAD, with gain of FOXA1-binding sites at NEPC regulatory elements and loss of FOXA1 at PRAD elements. FOXA1 exhibits features of a master transcriptional regulator. It is encompassed by a super-enhancer in NEPC and is involved in core regulatory circuits with neuronal lineage TFs such as ASCL1. Future studies will be necessary to determine whether the shift in FOXA1-binding sites is required for neuroendocrine differentiation, or merely correlative. In either case, we establish FOXA1 as a dependency in NEPC. The finding that FOXA1 is essential in NEPC has perhaps been overlooked because candidate drivers of NEPC have been identified mainly based on differential transcript expression or somatic DNA alterations^6,10,28,29^. Our study demonstrates that epigenomic profiling can identify cancer dependencies that are difficult to detect based on mutational and transcriptional profiling alone.

A dependency of NEPC on FOXA1 is unexpected based on prior work. FOXA1 has been reported to *inhibit* neuroendocrine differentiation of prostate adenocarcinoma, based on the observations that FOXA1 is downregulated in NEPC and that FOXA1 knock-down induces neuroendocrine features in PRAD cell lines^30^. Our data demonstrate that FOXA1 remains crucial in NEPC despite consistent, modest transcript downregulation in NEPC compared to PRAD. Our H3K27ac HiChIP data reveal that in NEPC, *FOXA1* contacts distal super-enhancers that are distinct from its PRAD enhancers and contain binding sites for NE-associated TFs such as ASCL1 and INSM1 (Fig. 2d and Supplementary Fig. 5). Thus, an NEPC-specific regulatory program may maintain FOXA1 expression at lower levels that are conducive to NE gene expression, reconciling our findings with the reported pro-neuroendocrine effects of partial FOXA1 suppression in PRAD^30^. This hypothesis should be tested in future mechanistic studies. While our data show that FOXA1 is essential in NEPC, further studies are required to determine if FOXA1 cistrome reprogramming directly activates Ne-CREs and to assess its role dynamic lineage plasticity.

FOXA1 may have a more general role in controlling neuroendocrine differentiation. For example, in small cell lung cancer (SCLC), a neuroendocrine lung cancer variant that can emerge de novo or from *EGFR*-mutant lung adenocarcinoma after targeted kinase inhibition, *FOXA1* is highly expressed and encompassed by a super-enhancer^31^. We observe extensive H3K27 acetylation in SCLC cell lines specifically at Ne-CREs and NEPC-enriched FOXA1-binding sites, suggesting similar enhancer usage between in SCLC and NEPC (Supplementary Fig. 6), consistent with recent reports^28,32^. The large set of Ne-CREs and NEPC-enriched FOXA1-binding sites could aid the pathologic diagnosis of neuroendocrine differentiation, which can be challenging and relies on only a handful of markers. Ultimately, therapeutic targeting of FOXA1 and/or proteins that collaborate with or covalently modify this TF^33^ presents an attractive strategy as FOXA1 is a common vulnerability in both PRAD and NEPC.

## Methods

### Patient-derived xenograft and tissue specimens

LuCaP patient-derived xenografts (PDXs) have been described previously^9,34,35^ with the exception of LuCaP 208.1. LuCaP 208.1 was derived from treatment-emergent NEPC and demonstrates typical small cell histology. All LuCaP PDXs, including LuCaP 208.1 were derived from resected prostate cancer with explicit written consent of patient donors as described previously^9^ under a protocol approved by the University of Washington Human Subjects Division IRB (#39053). PDXs were generated in compliance with all relevant ethical regulations for animal testing and research under protocol #39053. The University of Washington Institutional Animal Care and Use Committee approved all animal procedures including generation and processing of PDXs, including LuCaP 208.1. Liver metastasis needle biopsy specimens were obtained from the Dana-Farber Cancer Institute Gelb Center biobank and were collected under a DFCI/Harvard Cancer Center IRB-approved protocol (01-045) with informed consent of patients. Metastases were reviewed by a clinical pathologist. The NEPC metastasis was obtained from a patient with de novo metastatic prostate adenocarcinoma after 17 months of androgen deprivation therapy with leuprolide and bicalutamide. Immunohistochemistry revealed staining for synaptophysin, chromogranin, and NKX3-1 (weak), and absence of RB1, AR, and PSA.

### Epigenomic profiling

#### chromatin immunoprecipitation in LuCaP PDXs

Frozen tissue (20–30 mg for histone mark ChIP and 50–80 mg for transcription factor ChIP) was pulverized using the CryoPREP dry impactor system (Covaris). The tissue was then fixed using 1% formaldehyde (Thermo fisher) in phosphate-buffered saline (PBS) for 18 min either at 37 degrees Celsius (histone mark ChIP) or at room temperature (transcription factor ChIP) and was quenched with 125 mM glycine. Chromatin was lysed in ice-cold lysis buffer (50 mM Tris, 10 mM EDTA, 1% SDS with protease inhibitor for histone mark ChIP; 0.1% SDS, 0.5% sodium deoxycholate and 1% NP-40 with protease inhibitor for transcription factor ChIP) and was sheared to 300–800 bp using the Covaris E220 sonicator (105 watt peak incident power, 5% duty cycle, 200 cycles/burst for 10 min for histone mark ChIP; 140 watt peak incident power, 5% duty cycle, 200 cycles/burst for 20 min for transcription factor ChIP). Five volumes of dilution buffer (1% Triton X-100, 2 mM EDTA, 150 mM NaCl, 20 mM Tris-HCl pH 8.1) were added to chromatin for histone mark ChIP. The sample was then incubated with antibodies (H3K27ac, Diagenode, C15410196; H3K27me3, Cell Signaling 9733S; H3K4me3, Diagenode C15410003 premium; FOXA1, ab23738, Abcam) coupled with protein A and protein G beads (Life Technologies) at 4 degrees Celsius overnight. The chromatin was washed with RIPA wash buffer (100 mM Tris pH 7.5, 500 mM LiCl, 1% NP-40, 1% sodium deoxycholate) for 10 min six times and rinsed with TE buffer (pH 8.0) once. DNA was purified using MinElute column followed by incubation in the de-crosslinking buffer (1% SDS, 0.1 M NaHCO3 with Proteinase K and RNase A) at 65 degrees Celsius.

#### LNCaP ChIP

ChIP in LNCaP was performed according to published protocols^36^. Ten million cells were fixed with 1% formaldehyde at room temperature for 10 min and quenched. Cells were collected in lysis buffer (1% NP-40, 0.5% sodium deoxycholate, 0.1% SDS and protease inhibitor (#11873580001, Roche) in PBS)^37^. Chromatin was sonicated to 300–800 bp using a Covaris E220 sonicator (140 watt peak incident power, 5% duty cycle, 200 cycleburtst). Antibodies (FOXA1, ab23738, Abcam; H3K27ac, C15410196, Diagenode; ASCL1, ab74065) were incubated with 40 μl of Dynabeads protein A/G (Invitrogen) for at least 6 h before immunoprecipitation of the sonicated chromatin overnight. Chromatin was washed with LiCl wash buffer (100 mM Tris pH 7.5, 500 mM LiCl, 1% NP-40, 1% sodium deoxycholate) six times for 10 min sequentially.

#### ChIP sequencing

Sequencing libraries were generated from purified IP sample DNA using the ThruPLEX-FD Prep Kit (Rubicon Genomics). Libraries were sequenced using 150-base paired-end reads on an Illumina platform (Novogene).

#### ATAC-seq

LuCaP PDX tissues were resuspended and dounced in 300 ul of RSB buffer (10 mM Tris-HCl pH 7.4, 10 mM NaCl, and 3 mM MgCl_2_ in water) containing 0.1% NP-40, 0.1% Tween-20, and 0.01% digitonin. Homogenates were transferred to a 1.5 ml microfuge tube and incubated on ice for 10 min. Nuclei were filtered through a 40 μm cell strainer and nuclei were washed with RSB buffer and counted. Fifty thousand nuclei were resuspended in 50 μl of transposition mix^38^ (2.5 μl transposase (100 nM final), 16.5 μl PBS, 0.5 μl 1% digitonin, 0.5 μl 10% Tween-20, and 5 μl water) by pipetting up and down six times. Transposition reactions were incubated at 37 °C for 30 min in a thermomixer with shaking at 1000 r.p.m. Reactions were cleaned with Qiagen columns. Libraries were amplified^39^ and sequenced on an Illumina Nextseq 500 with 35 base paired-end reads.

### ChIP-seq data analysis

ChIP-sequencing reads were aligned to the human genome build hg19 using the Burrows-Wheeler Aligner (BWA) version 0.7.17^40^. Non-uniquely mapping and redundant reads were discarded. MACS v2.1.1.20140616^41^ was used for ChIP-seq peak calling with a *q*-value (FDR) threshold of 0.01. ChIP-seq data quality was evaluated by a variety of measures, including total peak number, FrIP (fraction of reads in peak) score, number of high-confidence peaks (enriched >10-fold over background), and percent of peak overlap with DNAse hypersensitivity (DHS) peaks derived form the ENCODE project. ChIP-seq peaks were assessed for overlap with gene features and CpG islands using annotatr^42^. IGV v2.8.2^43^ was used to visualize normalized ChIP-seq read counts at specific genomic loci. ChIP-seq heatmaps were generated with deepTools v3.3.1^44^ and show normalized read counts at the peak center ±2 kb unless otherwise noted. Overlap of ChIP-seq peaks was assessed using BEDTools v2.26.0. Peaks were considered overlapping if they shared one or more base pairs.

### Identification and annotation of PRAD- and NEPC-enriched ChIP-seq peaks

Sample–sample clustering, principal component analysis, and identification of lineage-enriched peaks were performed using Cobra v2.0^45^ (https://bitbucket.org/cfce/cobra/src/master/), a ChIP-seq analysis pipeline implemented with Snakemake^46^. ChIP-seq data from PRAD and NEPC LuCaP PDXs were compared to identify H3K27ac, H3K27me3, and FOXA1 peaks with significant enrichment in the NEPC or PRAD lineage. Only LuCaP PDXs from distinct patients were included, with the exception of the H3K27me3 differential peak analysis, which included both LuCaP 145.1 and 145.2, two LuCaP PDXs derived from distinct NEPC metastases from a single patient. A union set of peaks for each histone modification or TF was created using BEDTools. narrowPeak calls from MACS were used for H3K27ac and FOXA1, while broadPeak calls were used for H3K27me3. The number of unique aligned reads overlapping each peak in each sample was calculated from BAM files using BEDtools. Read counts for each peak were normalized to the total number of mapped reads for each sample. Quantile normalization was applied to this matrix of normalized read counts. Using DEseq2 v1.14.1^47^, lineage-enriched peaks were identified at the indicated FDR-adjusted *p*-value (*p*_adj_) and log_2_ fold-change cutoffs (H3K27ac, *p*_adj_ < 0.001, |log_2_ fold-change| >3; FOXA1, *p*_adj_ < 0.001, |log_2_ fold-change| >2; H3K27me3, *p*_adj_ < 0.01, |log_2_ fold-change| > 1). Unsupervised hierarchical clustering was performed based on Spearman correlation between samples. Principal component analysis was performed using the prcomp R function. Enriched de novo motifs in differential peaks were detected using HOMER version 4.7. The top non-redundant motifs were ranked by adjusted *p*-value.

The GREAT tool^48^ (V3.0) was used to asses for enrichment of Gene Ontology (GO) and MSigDB perturbation annotations among genes near differential ChIP-seq peaks, assigning each peak to the nearest gene within 500 kb. The cistromedb toolkit (http://dbtoolkit.cistrome.org/) was used to compare ChIP-seq peaks for overlap with peaks from a large database of uniformly analyzed published ChIP-seq data (quantified as a “GIGGLE score”)^49^. Published TFs and histone marks were ranked by similarity to the querry dataset based on the top 1000 peaks in each published dataset. Prior to cistromedb toolkit analysis, ChIP-seq peaks were mapped from hg19 to hg38 using the UCSC liftover tool (https://genome.ucsc.edu/cgi-bin/hgLiftOver).

For analysis of H3K27 acetylation in lung cancer at lineage-enriched candidate regulatory elements, fastq files were generated from sequence read archives (SRA) from published ChIP-seq experiments for SCLC^50^ and LUAD^51,52^ (SRA numbers SRR568435, SRR3098556, SRR4449027, SRR4449025, and SRR6124068).

For Fig. 5c, H3K27ac ChIP-seq peaks from primary peripheral blood monocytes (ENCFF540CVX) and epithelial keratinocytes (ENCFF943CBQ)^53^ were used as a comparator to peaks derived from LuCaP PDXs. For these comparisons, monocyte and keratinocyte peaks within 1 kb of a LuCaP peak were excluded.

### RNA-seq and differential expression analysis

RNA-seq data from human adenocarcinoma and NEPC have been reported previously^5^ and were obtained from dbGaP (accession number phs000909.v1.p1). Transcriptomes were sequenced from two replicates from each of five PRAD LuCaP PDXs (23, 77, 78, 81, and 96) and five NEPC LuCaP PDXs (49, 93, 145.1, 145.2, and 173.1). RNA concentration, purity, and integrity were assessed by NanoDrop (Thermo Fisher Scientific Inc.) and Agilent Bioanalyzer. RNA-seq libraries were constructed from 1 μg total RNA using the Illumina TruSeq Stranded mRNA LT Sample Prep Kit according to the manufacturer’s protocol. Barcoded libraries were pooled and sequenced on the Illumina HiSeq 2500 generating 50 bp paired-end reads. FASTQ files were processed using the VIPER workflow^54^. Read alignment to human genome build hg19 was performed with STAR v 2.7.0f^55^. Cufflinks was used to assemble transcript-level expression data from filtered alignments^56^. Differential gene expression analysis (NEPC vs. PRAD) was conducted using DESeq2^47^.

### H3K27ac HiChIP

Pulverized frozen tissue from LuCaP 173.1 was fixed with 1% formaldehyde in PBS at room temperature for 10 min^36^. Sample was incubated in lysis buffer and digested with MboI (NEB) for 4 h. After 1 h of biotin incorporation with biotin dATP, the sample was ligated using T4 DNA ligase for 4 h. Chromatin was sheared using 140 PIP, 5% duty cycle, and 200 cycles/burst for 8 min in shearing buffer composed of 1% NP-40, 0.5% sodium deoxycholate, and 0.1% SDS in PBS (LNCaP) or using 100 PIP, 5% duty cycle, 200 cycles/burst for 3 min in 1% SDS, 50 mM Tris (pH 8.1), and 5 mM EDTA (LuCaP 173.1). ChIP was then performed using H3K27Ac antibody (Diagenode, C1541019)^57^.

Immunoprecipitated sample was pulled down with streptavidin C1 beads (Life Technologies) and treated with Transposase (Illumina). Amplification was performed for the number of cycles required to reach 1/3 of the maximal fluorescence on quantitative PCR (qPCR) plot with SYBR® Green I(Life Technologies). Libraries were sequenced using 150-base paired-end reads on the Illumina platform (Novogene).

### Alignment and filtering using HiC-Pro

We processed paired-end fastq files using HiC-Pro^58^ to generate intra- and inter-chromosomal contact maps. The reads were first trimmed to remove adaptor sequences using Trim Galore (https://github.com/FelixKrueger/TrimGalore). Default settings from HiC-Pro were used to align reads to the hg19 human genome, assign reads to MboI restriction fragments, and remove duplicate reads. Only uniquely mapped valid read pairs involving two different restriction fragments were used to build the contact maps.

### FitHiChIP

We applied FitHiChIP^59^ for bias-corrected peak calling and DNA loop calling.

We used MACS2 broadPeak peak calls from H3K27ac ChIP-seq in LuCaP 173.1 (NEPC). 44,609 peaks were called at a *q*-value < 0.01. We used a 5 kb resolution and considered only interactions between 5 kb and 3 Mb. We used peak-to-peak (stringent) interactions for the global background estimation of expected counts (and contact probabilities for each genomic distance), and peak-to-all interactions for the foreground, meaning at least one anchor must overlap a H3K27ac peak. The corresponding FitHiChiP options specified are “IntType = 3” and “UseP2PBackgrnd = 1”.

### Assignment of enhancer-promoter interactions using H3K27ac HiChIP data

NCBI RefSeq genes (hg19) were downloaded from the UCSC genome table browser (https://genome.ucsc.edu/cgi-bin/hgTables). Only uniquely mapping genes were considered. The longest transcript was selected for genes with multiple annotated transcripts. We searched for H3K27ac HiChIP loops with one anchor (defined with a 5 kb window) overlapping a region between 0 and 5 kb upstream of a gene transcriptional start site. We selected subset of these loops for which the second anchor (with a 5 kb window) overlapped with H3K27ac peaks identified by ChIP-seq in LuCaP 173.1 (NEPC) or with NEPC-enriched H3K27ac peaks (Ne-CREs). Gene promoters and distal H3K27ac peaks/Ne-CREs were considered looped if each overlapped with an anchor of the same high-confidence H3K27ac HiChIP loop(s). To examine the association of regulatory element looping with gene expression, genes were binned by the number of distinct, looped Ne-CREs or H3K27ac peaks. Differential expression between NEPC and PRAD LuCaP PDXs, as assessed by DESeq2 analysis of LuCaP RNA-seq data, was plotted for genes in each bin. Wilcoxon rank-sum *p*-values were calculated for differential expression of genes looped to one versus two or more H3K27ac/Ne-CRE peaks. A *p*-value < 0.01 was considered significant.

### Master transcription factor analysis

#### Super-enhancer ranking analyses

Enhancer and super-enhancer (SE) calls were obtained using the Rank Ordering of Super-enhancer (ROSE2) algorithm^11^. We selected SEs assigned to transcription factors (TFs)^60,61^, and for each sample, we obtained the ranks of all TF SEs. Considering only the top 5% TFs by median ranking in NEPC or PRAD, we applied a one-sided Mann–Whitney *U*-test to identify lineage-enriched TF SEs (FDR = 10%).

#### Clique enrichment and clustering analysis

Clique enrichment scores (CESs) for each TF were calculated using clique assignments from Coltron^62^. Coltron assembles transcriptional regulatory networks (cliques) based on H3K27 acetylation and TF-binding motif analysis. The clique enrichment score for a given TF is the number of cliques containing the TF divided by the total number of cliques. We incorporated ATAC-seq data to restrict the motif search to regions of open chromatin. Using the CES, we performed clustering (distance = Canberra, agglomeration method = ward.D2) considering only TFs that appear in cliques in at least 80% of the samples in at least one lineage group (4 out of 5 NEPC and 11 out of 14 PRAD).

#### Motif enrichment at super-enhancers with loops to the FOXA1 locus

H3K27ac HiChIP data were used to select distal SEs that form three-dimensional contacts with the *FOXA1* locus. We used the Coltron algorithm to search for TF motifs in ATAC-seq peaks within these SEs. We considered all TFs that were categorized as expressed by Coltron based on H3K27ac levels at the TF gene locus. Motif enrichment for a TF was calculated as the total number of non-overlapping base pairs (bp) covered by the TF motif, divided by the summed length (in bp) of the SEs. Values in the heatmap legend correspond to percent coverage (i.e., the largest value corresponds to 0.4%).

### FOXA1 mutational profiling

*FOXA1* mutational status was assessed from exome sequence data (62x–110X depth of coverage). Each LuCaP PDX was sequenced using the Illumina Hi-seq platform with 100 bp paired-end reads. Hybrid capture was performed SeqCapV3. Mouse genome subtraction was performed using the mm10 genome build and reads were aligned to human reference genome hg19. For sequence analysis, bam files processed as per Genome Analysis Toolkit (GATK) best practice guideline^63^. We Used MuTect2 and HaplotypeCaller for mutation calls. All mutations were manually reviewed and subsequently annotated using Annovar^64^. Copy number was derived using the Sequenza R package^65^. Factera^66^ was used to predict structural events involving FOXA1.

### FOXA1 siRNA knock-down

WCM154 organoids were cultured and maintained as described^18^. Organoids were dissociated to single cells using TrypLE (ThermoFisher). One million cells were resuspended in 20 μl of electroporation buffer (BTXpress) and mixed with 60 pmole of control or FOXA1 On-target pool siRNA (Dharmacon). Then organoid-siRNA mixtures were transferred to a 16-well NucleocuvetteTM Strip and nucleofection was performed in a 4D-Nucleofector (Lonza). Following nucleofection, 10^5^ organoids cells were grown in a 12-well plate coated with 1% collagen I (ThermoFisher) for 7 days. Both adherent and floating cells were collected and stained with 0.4% trypan blue solution (ThermoFisher). Total cell numbers were measured by a hemocytometer. Cell proliferation with FOXA1 knock-down was normalized to control siRNA cells.

### FOXA1 shRNA knock-down

LNCaP, LNCaP 42D, and LNCaP 42F cells were seeded in parallel 6-well plates at 500, 500, or 100 k, respectively. Twenty-four hours later, cells were infected with lentivirus containing shRNAs targeting GFP control or *FOXA1*. Forty-eight hours following infection, equal cell numbers were seeded, and proliferation was assayed 6 days later using a Vi-Cell. Seventy-two hours following infection, a second plate infected in parallel was harvested for immunoblotting. The target sequence against GFP was CCACATGAAGCAGCACGACTT (shGFP). The target sequences against FOXA1 were GCGTACTACCAAGGTGTGTAT (shFOXA1-1) and TCTAGTTTGTGGAGGGTTAT (shFOXA1-2).

### FOXA1 CRISPR-Cas9 knock-out

Blasticidin-resistant Cas9-positive LNCaP, LNCaP 42D, and LNCaP 42F cells were cultured in 20 μg/ml blasticidin (Thermo Fisher Scientific, NC9016621) for 72 h to select for cells with optimal Cas9 activity. LNCaP, LNCaP 42D, and LNCaP 42F cells were seeded in parallel 6-well plates at 300, 300, 300, or 60 k, respectively. Cells were infected after 24 h with lentiviruses expressing sgRNAs targeting GFP control or *FOXA1*. Cells were subject to puromycin selection and harvested for immunoblot after 3 days. Six days following selection, cell viability was determined using a Vi-Cell. The target sequences against GFP were AGCTGGACGGCGACGTAAA (sgGFP1) and GCCACAAGTTCAGCGTGTCG (sgGFP2). The target sequences against FOXA1 were GTTGGACGGCGCGTACGCCA (sgFOXA1-1), GTAGTAGCTGTTCCAGTCGC (sgFOXA1-2), CAGCTACTACGCAGACACGC (sgFOXA1-3), and ACTGCGCCCCCCATAAGCTC (sgFOXA1-4).

### Western blots

For WCM154 western blots, cell pellets were lysed in RIPA buffer (MilliporeSigma, 20–188) supplemented with Protease/Phosphatase Inhibitor Cocktail (Cell Signaling Technology, 5872S). Protein concentrations were assayed with a Pierce BCA Protein Assay Kit (Thermo Fisher Scientific, PI23225), and protein was subsequently denatured in NuPAGE LDS sample buffer (Thermo Fisher Scientific, NP0007) containing 5% β-Mercaptoethanol. Thirteen micrograms of each protein sample was loaded onto NuPAGE 4–12% Bis-Tris Protein gels (Thermo Fisher Scientific), and samples were run in NuPAGE MOPS SDS Running Buffer (Thermo Fisher Scientific, NP0001). Following electrophoresis, proteins were transferred to nitrocellulose membranes via an iBlot apparatus (Thermo Fisher Scientific). After blocking in Odyssey Blocking Buffer (LI-COR Biosciences, 927-70010) for 1 h at room temperature, membranes were cut and incubated in primary antibodies diluted 1:1000 in Odyssey Blocking Buffer overnight at 4 °C. The next morning, membranes were washed three times with phosphate-buffer saline, 0.1% Tween (PBST) and then incubated with fluorescent anti-rabbit secondary antibodies (Thermo Fisher Scientific, NC9401842) for 1 h at room temperature. Membranes underwent five PBST washes and were then imaged using an Odyssey Imaging System (LI-COR Biosciences). Primary antibodies used include FOXA1 (Cell Signaling Technology, 58613S) and β-actin (Cell Signaling Technology, 8457L).

For LNCaP, LNCaP 42D, and LNCaP 42F western Blots, cell lysate was extracted using RIPA lysis buffer (Sigma) containing protease inhibitor (Roche) and phosphatase inhibitor (ThermoFisher). Fifty micrograms of protein was subjected to a 4–15% Mini-PROTEAN Precast electrophoresis gel (Bio-Rad) then transferred to 0.22 µm nitrocellulose membrane (Bio-Rad) and blocked in 5% blotting grade blocker (Bio-Rad). Membranes were incubated with primary antibodies overnight (FOXA1, Abcam, 1:2000, ab23738; Synaptophysin, Cell Marque, 1:5000, MRQ-40; INSM1, Santa Cruz, 1:2000, sc-377428; FOXA2, Abcam; 1:2500, ab108422; Chromogranin A, Abcam, 1:2000, ab15160; Vinculin, Cell signaling, 1:5000, #13901). Membranes were then washed in 1x Tris-buffered saline with 0.5% Tween-20 (Boston BioProducts) and incubated with secondary antibodies (mouse, Bio-Rad, 1:2500; rabbit, Bio-Rad, 1:2500). Western HRP substrate kit was used to detect chemiluminescent signal (Millipore, Classico).

### Analysis of FOXA1-binding sites across prostate cancer states

FOXA1 cistromes were compared across different states of prostate cancer progression (normal prostate, prostate-localized adenocarcinoma, PDXs derived from metastatic castration resistant prostate cancer, and PDXs derived from NEPC). FOXA1 ChIP from normal prostate tissue and prostate-localized adenocarcinoma will be reported separately (Pomerantz et al., submitted). For normal prostate tissue FOXA1 ChIP, tissue cores were obtained from regions of prostatectomy specimens with dense epithelium and no evidence of neoplasia on review by a genitourinary pathologist. PDX samples used are listed in Supplementary Data 1. PDXs derived from localized prostate cancer were excluded from this analysis. As the normal prostate and localized adenocarcinoma samples were sequenced with single-end sequencing with an average of ~20 million reads, paired-end sequencing data from LuCaP PDXs were down-sampled to 20 M reads, using a single-end trimmed to 75 base pairs using seqtk (https://github.com/lh3/seqtk).

Pairwise comparisons were made between normal prostate (*N* = 5) and localized PRAD (*N* = 5), localized PRAD and metastatic PRAD PDXs (*N* = 11), and metastatic PRAD PDXs and NEPC PDXs (*N* = 5) using DESeq2 as described above. Peaks were considered significantly different between groups at a log2 |fold-change| threshold of 2 and FDR-adjusted *p*-value threshold of 0.001. “Shared” peaks were defined as the intersection of all peaks that were present in each group but not significantly different in any comparison.

### Immunohistochemistry

Immunohistochemistry was performed on tissue microarray (TMA) sections. TMA slides were stained for FOXA1 (Abcam ab170933, 1:100 dilution with 10 mM NaCitrate antigen retrieval) and FOXA2 (Abcam ab108422, 1:500 dilution with 10 mM NaCitrate antigen retrieval) using a standard procedure^67^. Rabbit IgG was used as a negative control. Nuclear staining intensity was assigned levels 0, 1+, 2+, or 3+ and H-scores were calculated as: [1 x (% of 1+ cells) + 2 x (% of 2+ cells) + 3 x (% of 3+ cells)]. Evaluations were performed in a blinded fashion.

### ASCL1/NKX2-1 overexpression in LNCaP

#### Transduction of LNCaP cells with ASCL1 and NKX2-1

The open reading frames of ASCL1 and NKX2-1 were cloned into the pLX_TRC302 lentiviral expression vector (Broad Institute) using the gateway recombination system. A construct expressing eGFP (pLX_TRC302_GFP) was used as a negative control. Viruses were generated by transfecting 293T cells with packaging vectors pVsVg and pDelta8.9. Supernatant was collected after 48 h. LNCaP cells were transduced in the presence of 4 μg/ml polybrene and harvested after 3 days for RNA-seq, ATAC-seq, and ChIP-seq.

ChIP seq was performed as described above, using 10–15 million cells fixed with 1% paraformaldehyde for 10 min at room temperature, followed by quenching with glycine. RNA was isolated using QIAGEN RNeasy Plus Kit and cDNA synthesized using Clontech RT Advantage Kit. Quantitative PCR was performed on a Quantstudio 6 using SYBR green. Primers used for quantitative reverse transcription PCR (qRT-PCR) are listed in Supplementary Table 4.

### Analysis of promoter H3K4 and H3K27 trimethylation

Refseq gene coordinates (hg19) were compiled, selecting the longest isoform where multiple were annotated. Normalized tag counts from H3K27me3 and H3K4me3 ChIP-seq within 2 kb of each transcriptional start site (TSS) were calculated for each sample, then averaged across multiple samples in each group (five NEPC PDXs, five PRAD PDXs, three normal prostates; Pomerantz et al., submitted). Contours were calculated using the R function geom_density_2d from the ggplot2 package; they represent the 2d kernel density estimation for all included transcriptional start sites. Gene promoters were assigned “active”, “bivalent”, “unmarked”, and “repressed” annotations based on H3K4me3 and H3K27me3 levels. High/low cutoffs for these marks were determined as follows. First, the H3K4me3 normalized tag counts near each TSS were fit to two normal distributions using the normalmixEM R function from the mixtools R package. The cutoff between H3K4me3-high and -low was set at four standard deviations below the mean value of the H3K4me3-high distribution. Next, the normalized H3K27me3 tag counts near H3K4me3-high TSSs were fit to two normal distributions. The cutoff for H3K27me3-high promoters was set at four standard deviations above the mean value of the H3K27me3-low distribution. The Pearson Chi-squared test was used to quantify significance of enrichment of NEPC-upregulated genes in the “bivalent” quadrant compared to “repressed” or “unmarked” quadrants. NEPC-upregulated genes were defined as those with log_2_ fold-change >3 and adjusted *p*-value < 1 x 10^−6^ in NEPC vs. PRAD. The results of the analysis were robust to using other *p*-value and differential expression thresholds.

### Methylation analysis of normal prostate

Whole-genome bisulfite sequencing data from histologically normal prostate tissue were reported previously^68^ and processed in our prior report^69^. Briefly, bases with a Phred score below 20 were trimmed and adapter sequences were discarded using Trim Galore! (version 0.4.4_dev; https://github.com/FelixKrueger/TrimGalore). Trimmed reads were mapped to the hg19 reference genome using Bowtie2 and Bismark v0.19.0^70^. Methylated read counts and total coverage for CpGs were extracted using the bismark_methylation_extractor command and portions of methylated CpGs were computed with the bsseq Bioconductor package^71^. CpG methylation at indicated sites was visualized using deepTools^44^ v3.3.1.

### Statistical tests

All statistical tests were two-sided except where otherwise indicated.

### Reporting summary

Further information on research design is available in the Nature Research Reporting Summary linked to this article.

## Supplementary information

Supplementary Information

Description of Additional Supplementary Files

Supplementary Data 1–4

Reporting Summary

## Data Availability

The ChIP-seq data generated in this study (sequencing reads in fastq format and normalized read counts in bigwig format) have been deposited in GEO under accession number GSE161948. The RNA-seq data from clinical NEPC are available in dbGaP under accession code phs000909.v.p1. scRNAseq data used in this study are available at in GEO under accession number GSE120716. Lung cancer H3K27ac ChIP-seq data are available in SRA under accession numbers SRR568435, SRR3098556, SRR4449027, SRR4449025, and SRR6124068. H3K27ac ChIP-seq peaks from primary peripheral blood monocytes and keratinocytes are available from ENCODE (https://www.encodeproject.org/) under accession numbers ENCFF540CVX and ENCFF943CBQ. The WGBS data used in this study were obtained from the authors and are available on request by contacting Dr. Jianhua Luo at the Department of Pathology of the University of Pittsburgh (luoj@msx.upmc.edu)^68^. LuCaP PDXs are available upon request from Dr. Eva Corey (ecorey@uw.edu). Source data are provided with this paper.

## Source data

Source Data

## Code availability

Code for the Cobra pipeline^45^ used for the analyses in this paper is available at https://bitbucket.org/cfce/cobra/src/master/.
