# Supplementary material for: Reprogramming of the FOXA1 cistrome in treatment-emergent neuroendocrine prostate cancer
Source: Nat Commun. 2021 Mar 30;12:1979. doi: 10.1038/s41467-021-22139-7 (PMC8010057; doi:10.1038/s41467-021-22139-7)
Supplement: Supplementary file 1 — Supplementary Information [file 41467_2021_22139_MOESM1_ESM.docx]

**Supplementary Table 1:** Gene Ontology annotations enriched among genes near NEPC-enriched candidate regulatory elements

| **Gene Ontology Term** | **Rank** | **P-Value** | **FDR Q-Value** | **Fold Enrich-ment** | **Observed Region Hits** | **Region Set Coverage** |
| --- | --- | --- | --- | --- | --- | --- |
| neuron fate specification | 28 | 6.19E-25 | 2.91E-22 | 2.63 | 151 | 1.01E-02 |
| noradrenergic neuron differentiation | 68 | 1.34E-16 | 2.59E-14 | 3.53 | 62 | 4.14E-03 |
| neuroepithelial cell differentiation | 76 | 8.16E-16 | 1.41E-13 | 2.22 | 128 | 8.54E-03 |
| sympathetic nervous system development | 115 | 4.89E-13 | 5.58E-11 | 2.18 | 108 | 7.21E-03 |
| regulation of the force of heart contraction | 167 | 5.31E-11 | 4.18E-09 | 2.40 | 72 | 4.80E-03 |
| adrenal gland development | 181 | 1.77E-10 | 1.28E-08 | 2.37 | 70 | 4.67E-03 |
| startle response | 182 | 1.97E-10 | 1.42E-08 | 2.12 | 88 | 5.87E-03 |
| positive regulation of gastrulation | 219 | 9.82E-10 | 5.89E-08 | 2.89 | 45 | 3.00E-03 |
| neuron cell-cell adhesion | 277 | 1.01E-08 | 4.78E-07 | 2.04 | 78 | 5.21E-03 |
| retina layer formation | 282 | 1.22E-08 | 5.70E-07 | 2.13 | 70 | 4.67E-03 |
| mechanosensory behavior | 326 | 7.35E-08 | 2.96E-06 | 2.15 | 61 | 4.07E-03 |
| pancreatic A cell differentiation | 355 | 1.67E-07 | 6.17E-06 | 3.38 | 26 | 1.74E-03 |
| maternal behavior | 389 | 4.03E-07 | 1.36E-05 | 2.31 | 46 | 3.07E-03 |
| positive regulation of protein exit from endoplasmic reticulum | 576 | 1.04E-05 | 2.37E-04 | 2.63 | 27 | 1.80E-03 |
| negative regulation of glial cell proliferation | 653 | 2.66E-05 | 5.36E-04 | 2.13 | 37 | 2.47E-03 |
| positive regulation of lipopolysaccharide-mediated signaling pathway | 804 | 1.38E-04 | 2.26E-03 | 2.14 | 30 | 2.00E-03 |
| regulation of cardiac muscle cell membrane potential | 1426 | 3.90E-03 | 3.60E-02 | 2.24 | 15 | 1.00E-03 |

**Supplementary Table 2:** Quantification of nuclear FOXA1/FOXA2 staining in LuCaPs by immunohistochemistry. Nuclear staining intensity was assigned levels 0, 1+, 2+, or 3+ and H-scores were calculated as: [1 x (% of 1+ cells) + 2 x (% of 2+ cells) + 3 x (% of 3+ cells)]. Evaluations were performed in a blinded fashion.

| **PDX** | **Histology** | **FOXA1 H-score** | **FOXA2 H-score** |
| --- | --- | --- | --- |
| LuCaP 23.1 | PRAD | 150.0 | 0.0 |
| LuCaP 23.1CR | PRAD | 150.0 | 0.0 |
| LuCaP 35 | PRAD | 200.0 | 0.0 |
| LuCaP 35CR | PRAD | 200.0 | 0.0 |
| LuCaP 58 | PRAD | 150.0 | 0.0 |
| LuCaP 70 | PRAD | 150.0 | 0.0 |
| LuCaP 70CR | PRAD | 150.0 | 0.0 |
| LuCaP 73 | PRAD | 183.3 | 0.0 |
| LuCaP 73CR | PRAD | 200.0 | 0.0 |
| LuCaP 77 | PRAD | 200.0 | 0.0 |
| LuCaP 77CR | PRAD | 200.0 | 0.0 |
| LuCaP 78 | PRAD | 200.0 | 0.0 |
| LuCaP 78CR | PRAD | 200.0 | 0.0 |
| LuCaP 81 | PRAD | 200.0 | 0.0 |
| LuCaP 81CR | PRAD | 200.0 | 0.0 |
| LuCaP 86.2 | PRAD | 200.0 | 0.0 |
| LuCaP 86.2CR | PRAD | 200.0 | 0.0 |
| LuCaP 92 | PRAD | 100.0 | 0.0 |
| LuCaP 93 | NEPC | 300.0 | 0.0 |
| LuCaP 96 | PRAD | 200.0 | 80.0 |
| LuCaP 96CR | PRAD | 200.0 | 71.7 |
| LuCaP 105 | PRAD | 300.0 | 73.3 |
| LuCaP 105CR | PRAD | 200.0 | 6.7 |
| LuCaP 136 | PRAD | 133.3 | 0.0 |
| LuCaP 136CR | PRAD | 200.0 | 0.0 |
| LuCaP 141 | PRAD | 150.0 | 0.0 |
| LuCaP 145.1 | NEPC | 150.0 | 226.7 |
| LuCaP 145.1 | NEPC | 150.0 | 286.7 |
| LuCaP 147 | PRAD | 200.0 | 0.0 |
| LuCaP 147CR | PRAD | 188.9 | 0.0 |
| LuCaP 167 | PRAD | 200.0 | 0.0 |
| LuCaP 167CR | PRAD | 200.0 | 0.0 |
| LuCaP 170.1 | PRAD | 150.0 | 0.0 |
| LuCaP 170.2 | PRAD | 246.7 | 0.0 |
| LuCaP 170.3 | PRAD | 250.0 | 47.8 |
| LuCaP 173.1 | NEPC | 200.0 | 12.8 |
| LuCaP 173.2A | PRAD | 168.8 | 223.3 |
| LuCaP 173.2B | PRAD | 200.0 | 241.1 |
| LuCaP 174.1 | PRAD | 183.3 | 0.0 |
| LuCaP 176 | PRAD | 188.9 | 0.0 |
| LuCaP 189.3 | PRAD | 116.7 | 0.0 |
| LuCaP 189.4 | PRAD | 150.0 | 0.0 |

**Supplementary Table 3**: FOXA1 mutational status of LuCaP PDXs.

| **PDX** | **Estimated tumor ploidy** | **Estimated copy number** | **Estimated absolute copy number** | **Mutation** | **Complex structural variant** |
| --- | --- | --- | --- | --- | --- |
| LuCaP 23.1 | 3.1 | 0 | 3 | None detected | None detected |
| LuCaP 23.1CR | 3.1 | 0 | 3 | None detected | None detected |
| LuCaP 35 | 3.1 | -1 | 1 | None detected | None detected |
| LuCaP 35CR | 2.9 | -1 | 1 | None detected | None detected |
| LuCaP 49 | 2.1 | -1 | 1 | None detected | None detected |
| LuCaP 58 | 3.4 | -1 | 2 | None detected | None detected |
| LuCaP 70 | 2.5 | 0 | 2 | None detected | None detected |
| LuCaP 70CR | 2.6 | 0 | 2 | None detected | None detected |
| LuCaP 73 | 3.7 | -1 | 2 | None detected | None detected |
| LuCaP 73CR | 3.8 | 0 | 2 | None detected | None detected |
| LuCaP 77 | 2.1 | 0 | 2 | p.S395ifdel | None detected |
| LuCaP 77CR | 2.1 | 0 | 2 | None detected | None detected |
| LuCaP 78 | 2.5 | 0 | 2 | None detected | None detected |
| LuCaP 78CR | 2.4 | 0 | 2 | None detected | None detected |
| LuCaP 81 | 2.3 | 0 | 2 | p.K264ifinsK | Hemizygous Inversion(chr14:37477643-38060842) Exon 1 SLC25A21 - Exon2 FOXA1 |
| LuCaP 81CR | 2.3 | 0 | 2 | p.K264ifinsK | Hemizygous Inversion(chr14:37477643-38060842) Exon 1 SLC25A21 - Exon2 FOXA1 |
| LuCaP 86.2 | 2.6 | 1 | 4 | None detected | None detected |
| LuCaP 86.2CR | 2.6 | 0 | 3 | None detected | None detected |
| LuCaP 92 | 3.2 | 0 | 3 | None detected | None detected |
| LuCaP 93 | 3 | 0 | 3 | None detected | None detected |
| LuCaP 96 | 3.2 | 0 | 2 | None detected | None detected |
| LuCaP 96CR | 3.3 | 0 | 3 | None detected | None detected |
| LuCaP 105 | 2.1 | 0 | 2 | None detected | None detected |
| LuCaP 105CR | 2 | -1 | 1 | None detected | None detected |
| LuCaP 136 | 1.9 | 0 | 2 | p.A353Qfs*6 | None detected |
| LuCaP 136CR | 1.8 | 0 | 2 | p.A353Qfs*6 | None detected |
| LuCaP 141 | 3.1 | 0 | 2 | p.R262 R265Ifdel | None detected |
| LuCaP 145.1 | 3.3 | -1 | 1 | None detected | None detected |
| LuCaP 145.2 | 3.2 | -1 | 1 | None detected | None detected |
| LuCaP 147 | 2.2 | 0 | 2 | None detected | None detected |
| LuCaP 147CR | 2.3 | 0 | 2 | None detected | None detected |
| LuCaP 167CR | 2.3 | 1 | 4 | p.S250 251ifins*8 | Hemizygous Inversion(chr14:35700959-38060809) Intragenic KIAA0391 - Exon2 FOXA1 |
| LuCaP 170.1 | 3.8 | 1 | 7 | None detected | None detected |
| LuCaP 170.2 | 3.8 | 1 | 7 | None detected | None detected |
| LuCaP 170.3 | 4.6 | 0 | 4 | None detected | None detected |
| LuCaP 173.1 | 2.3 | 2 | 5 | None detected | None detected |
| LuCaP 173.2 | 2.3 | 0 | 2 | None detected | None detected |
| LuCaP 174.1 | 3.5 | 0 | 3 | None detected | None detected |
| LuCaP 176 | 2.5 | 0 | 3 | None detected | None detected |
| LuCaP 189.3 | 2.7 | -1 | 1 | None detected | None detected |
| LuCaP 189.4 | 2.7 | 1 | 4 | None detected | None detected |

**Supplementary Table 4:** Primers used for qRT-PCR

| AR qRT-PCR fwd | GTGTCAAAAGCGAAATGGGC |
| --- | --- |
| AR qRT-PCR rev | GCTTCATCTCCACAGATCAGG |
| ASCL1 qRT-PCR fwd | CTACTCCAACGACTTGAACTCC |
| ASCL1 qRT-PCR rev | AGTTGGTGAAGTCGAGAAGC |
| GAPDH qRT-PCR fwd | CATGAGAAGTATGACAACAGCCT |
| GAPDH qRT-PCR rev | AGTCCTTCCACGATACCAAAGT |
| SOX2 qRT-PCR fwd | CACACTGCCCCTCTCAC |
| SOX2 qRT-PCR rev | TCCATGCTGTTTCTTACTCTCC |
| SYP qRT-PCR fwd | AGACAGGGAACACATGCAAG |
| SYP qRT-PCR rev | TCTCCTTAAACACGAACCACAG |


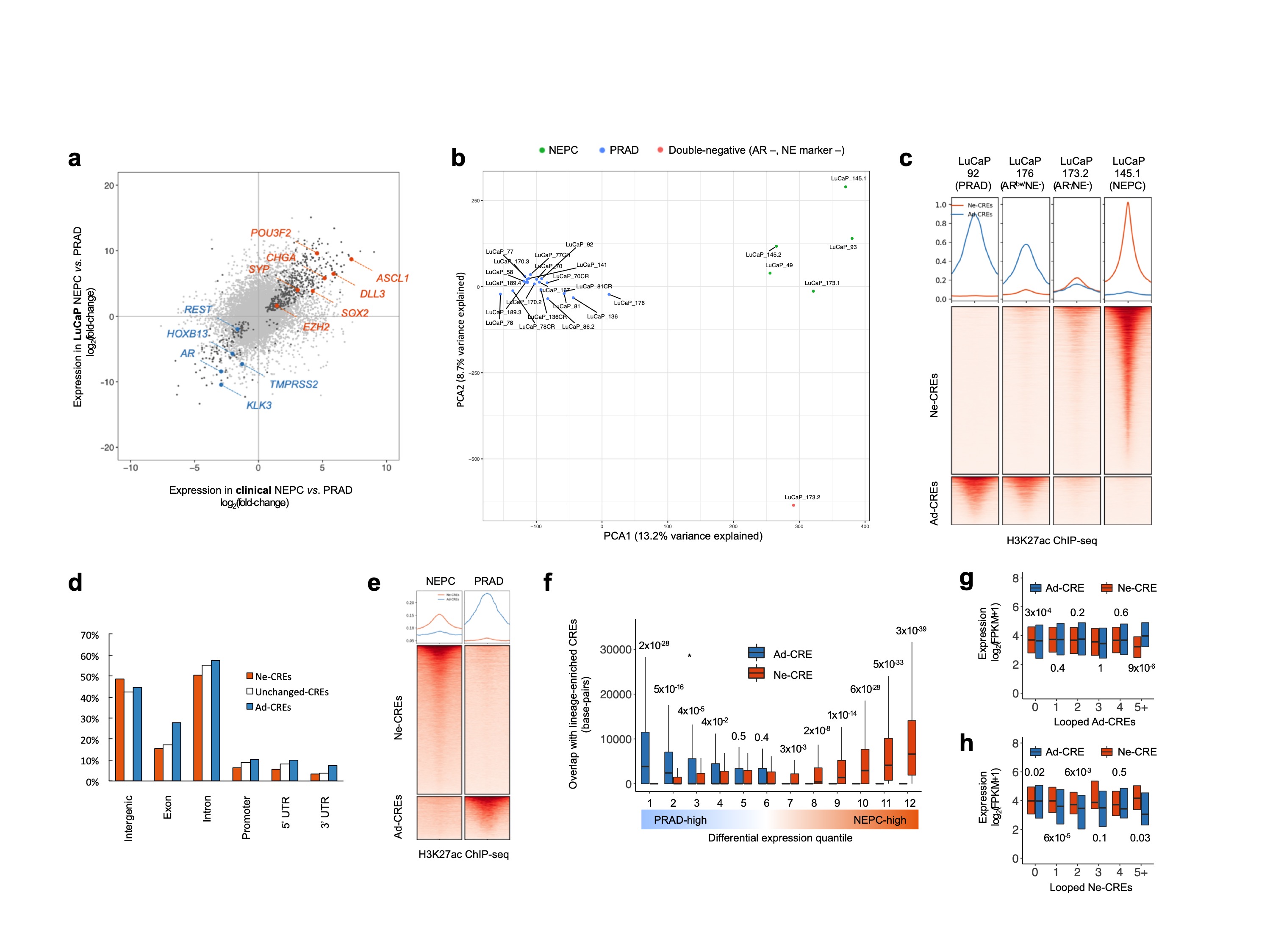


**Supplementary Figure 1. Epigenomic divergence of NEPC and PRAD. a**, Comparison of differential gene expression between PRAD and NEPC in LuCaP PDXs (5 of each lineage, with two replicates for each sample) and clinical prostate tumors^5^. Dark gray signifies genes with significant differential expression (two-sided p<10^-4^) in both PDXs and clinical tumors. **b**, Principal component analysis of PRAD and NEPC PDXs based on H3K27ac profiles. “DN” indicates a “double-negative” PDX lacking AR or NE marker expression. **c**, Normalized H3K27ac tag density for AR^-^/NE^-^ and AR^low^/NE^-^ PDX at Ne-CREs and Ad-CREs, compared to representative PRAD and NEPC PDXs. Profile plots (top) indicate the average tag density at Ne-CREs (orange) and Ad-CREs (blue). **d,** Genomic annotations for lineage-specific and shared H3K27ac peaks. **e**, Normalized H3K27ac tag density at Ne-CREs and Ad-CREs in a clinical NEPC liver metastasis and a PRAD liver metastasis. Profile plots (top) indicate the average tag density at Ne-CREs (orange) and Ad-CREs (blue). **f**, Overlap of Ne-CREs (n=14,985) and Ad-CREs (n=4,338) with a 200kb window centered around the transcriptional start sites of 17,603 genes ranked by differential expression in NEPC vs PRAD. Box boundaries correspond to 1^st^ and 3^rd^ quartiles; whiskers extend to a maximum of 1.5 x the inter-quartile range. **g-h**, expression of genes with the indicated number of distinct looped Ad-CREs (g) or Ne-CREs (h) detected by H3K27ac HiChIP in LuCaP 173.1 (NEPC). Box boundaries correspond to 1^st^ and 3^rd^ quartiles; whiskers extend to a maximum of 1.5 x the inter-quartile range. *p*-values for (f)-(h) were derived from two-sided Wilcoxon paired samples tests. Source data are provided as a Source Data file.


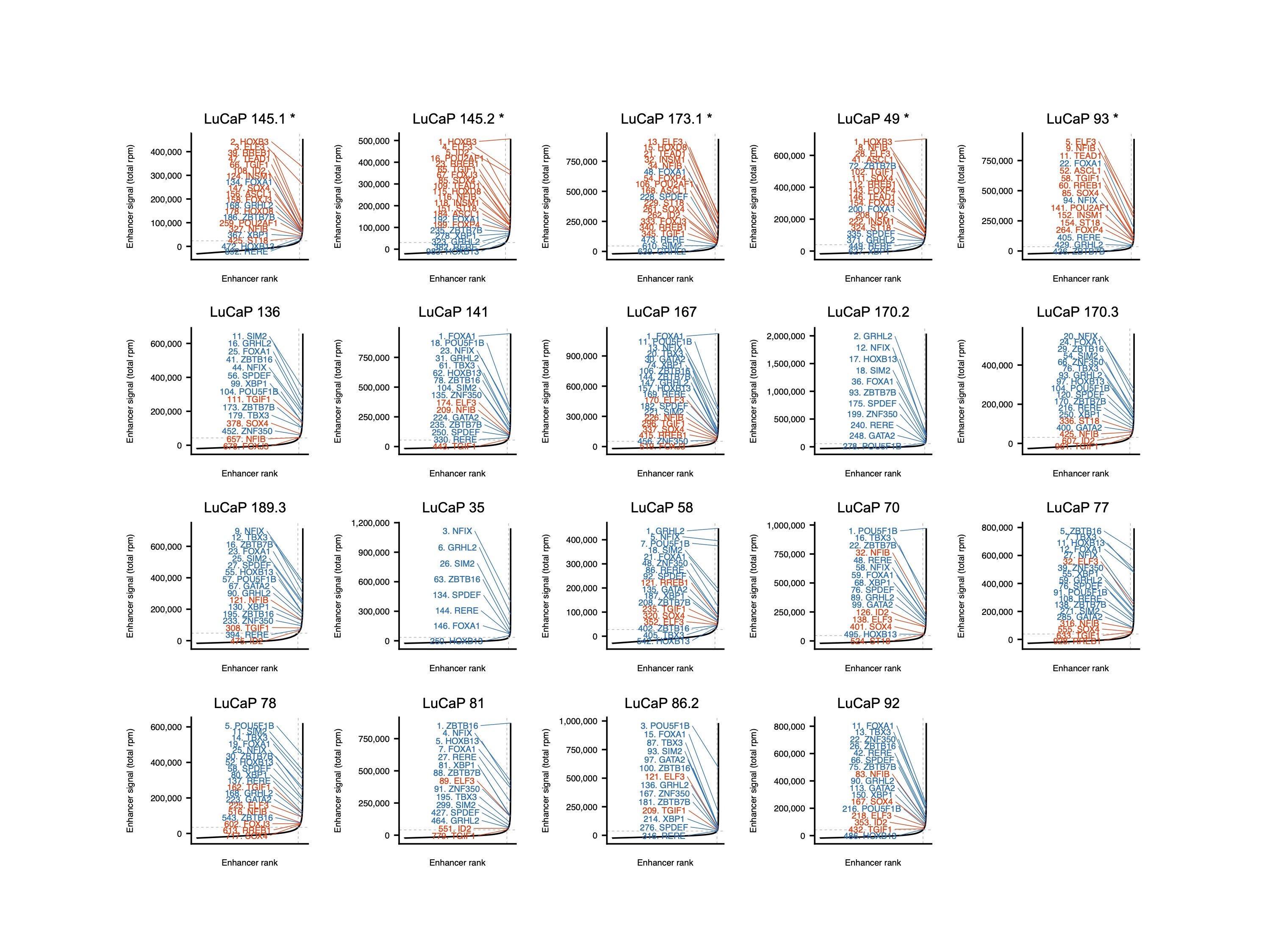


**Supplementary Figure 2.** **Super-enhancers (SEs) encompassing transcription factor genes that are differentially H3K27 acetylated in NEPC *vs.* PRAD**. SEs are ranked by H3K27ac signal. NEPC-enriched SEs are shown in orange; PRAD-enriched SEs are shown in blue (methods). Asterisk (*) indicates NEPC LuCaP PDXs.

**
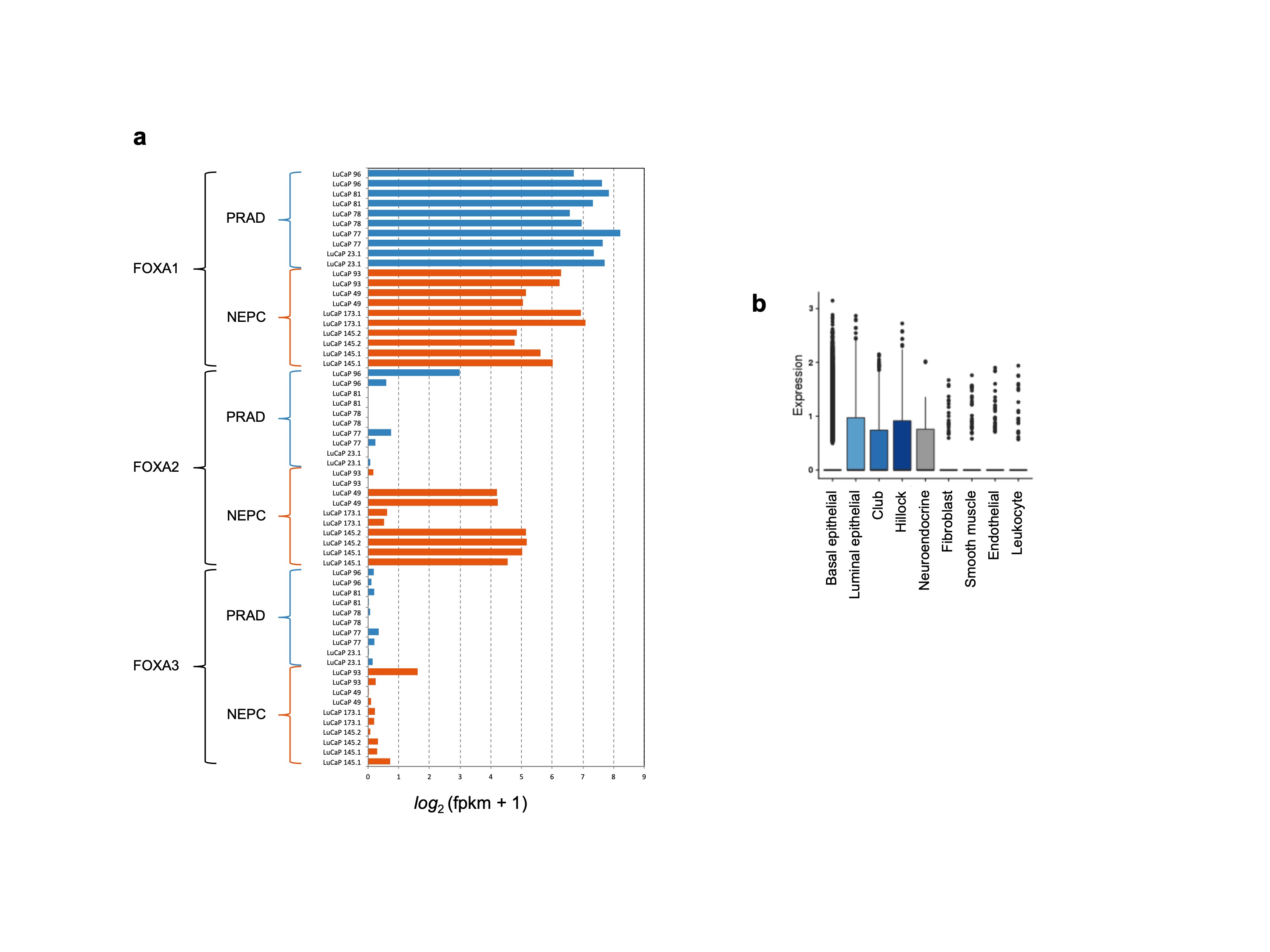
**

**Supplementary Figure 3.** **Expression of FOXA1 in PRAD, NEPC, and benign prostatic tissue**. **a**, Transcript expression of FOXA family members in 5 NEPC and 5 PRAD LuCaP PDXs (two replicates each) by RNA-seq. **b**, FOXA1 expression across benign prostate cell types in a published single-cell transcriptome sequencing dataset^72^. Box boundaries correspond to 1^st^ and 3^rd^ quartiles; whiskers extend to a maximum of 1.5 x the inter-quartile range.


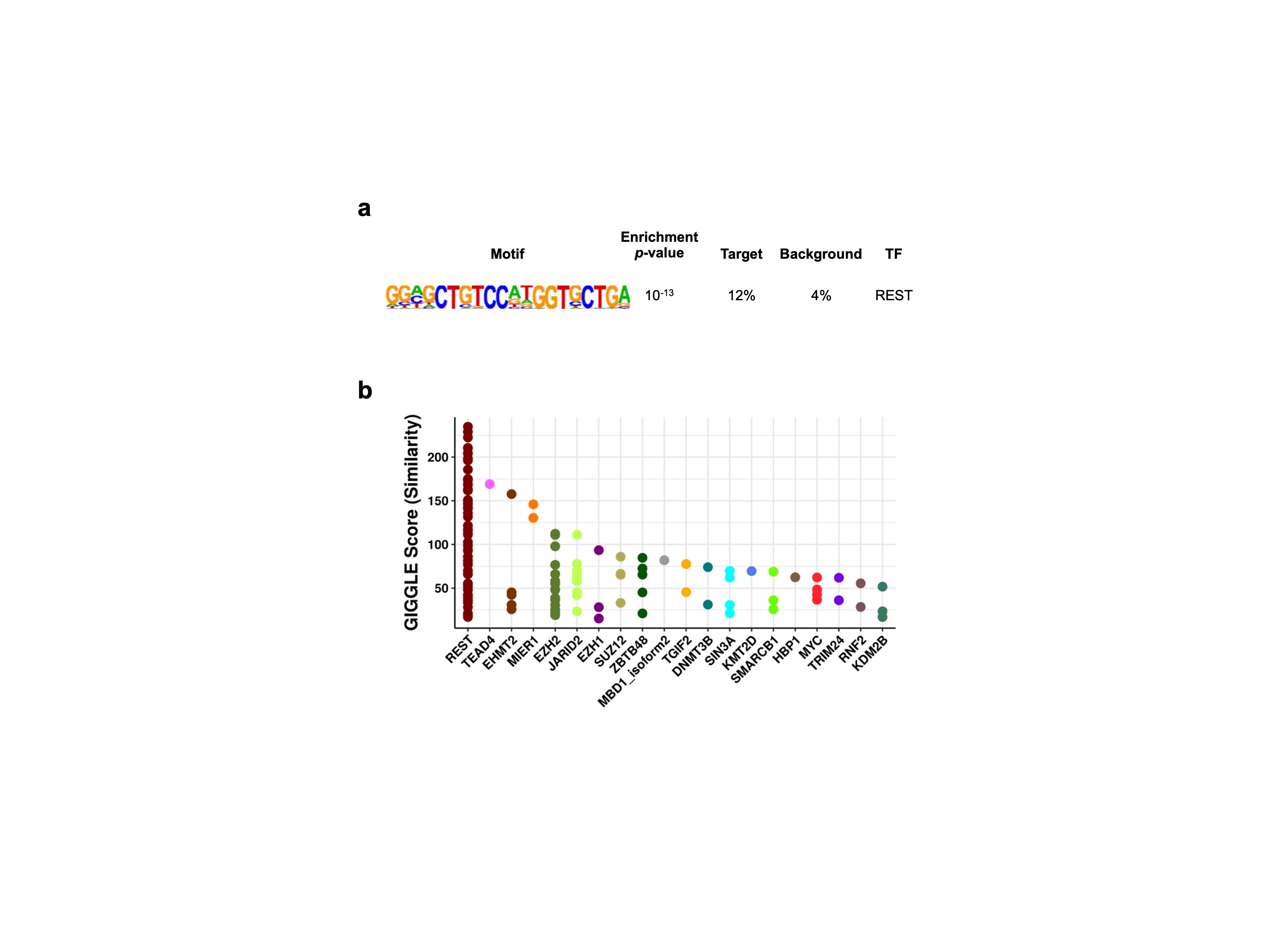


**Supplementary Figure 4. Motif enrichment and TF binding of differentially H3K27 trimethylated promoters. a,** Motif enrichment of promoters with diminished H3K27me3 in NEPC compared to PRAD. Only the indicated motif was significantly enriched. **b**, Cistromedb toolkit analysis of published ChIP-seq datasets (dbtoolkit.cistrome.org), ranked by their degree of overlap with the differentially H3K27 trimethylated promoters analyzed in **a**.

**
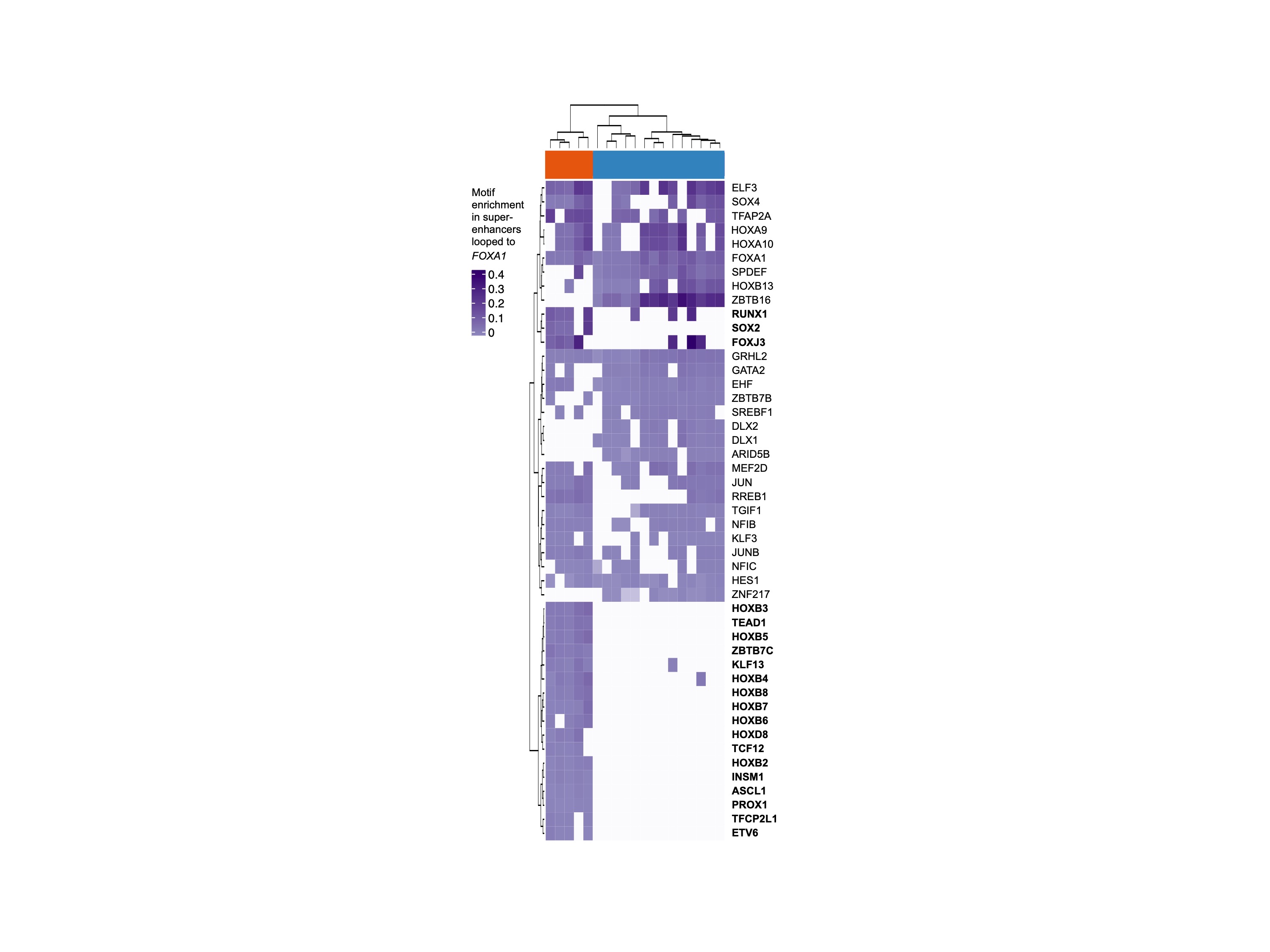
**

**Supplementary Figure 5.** Candidate TFs involved in regulation of the FOXA1 locus. Normalized abundance of TF binding motifs at superenhancers looped to the *FOXA1* locus, as assessed by H3K27ac Hi-ChIP in NEPC (LuCaP 173.1) and PRAD (LNCaP). TFs with motif enrichment primarily in NEPC are shown in bold.

**
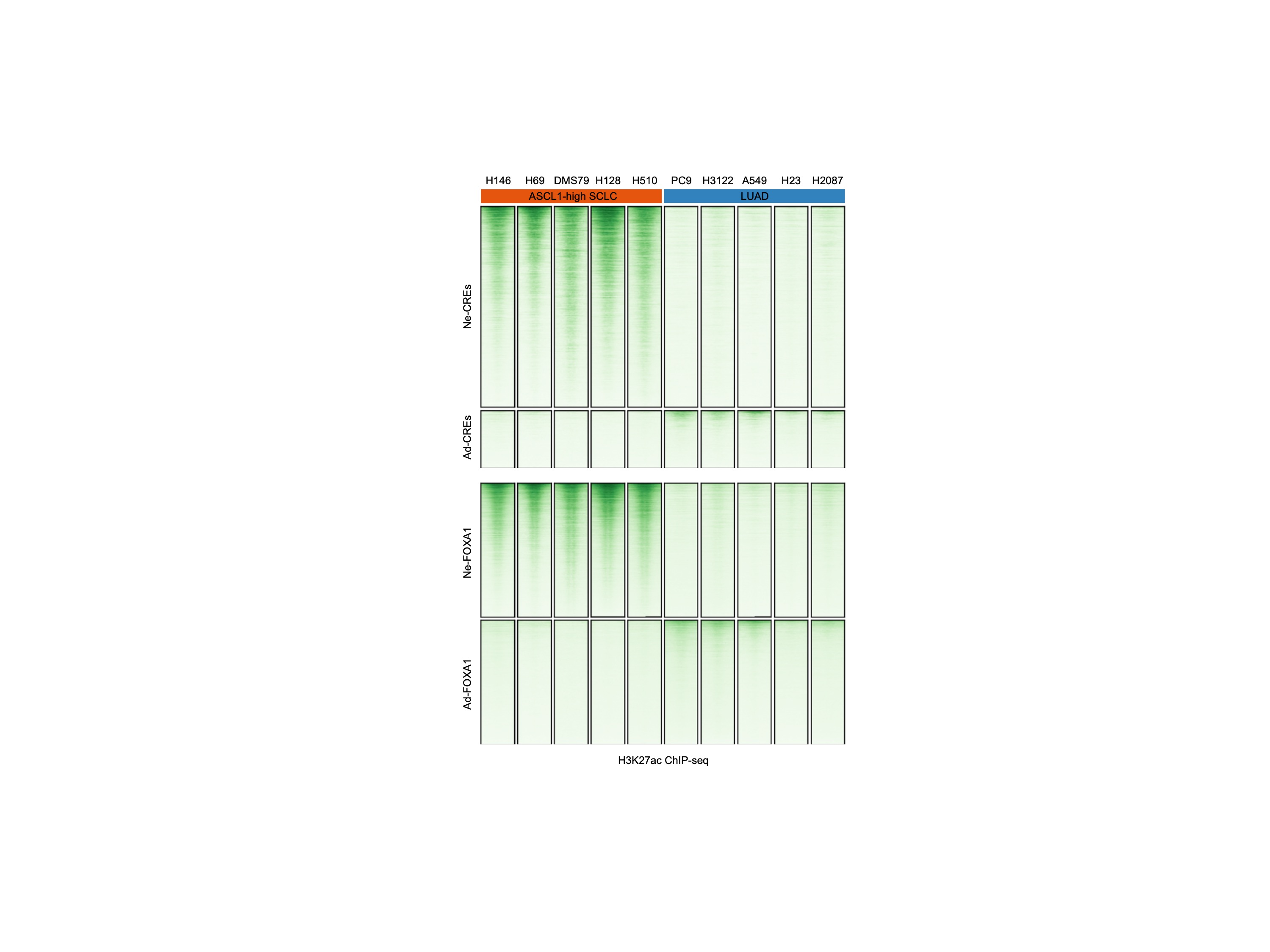
**

**Supplementary Figure 6. Activation of neuroendocrine candidate regulatory elements in small cell lung cancer.** H3K27ac ChIP-seq profiles of ASCL1-high small cell lung cancer (SCLC) cell lines at Ne-CREs and Ad-CREs (top) and at NEPC-enriched and PRAD-enriched FOXA1 binding sites (bottom). Five lung adenocarcinoma (LUAD) cell lines are shown for comparison. Data are from references^50-52^.
