# Supplementary material for: Reprogramming of the FOXA1 cistrome in treatment-emergent neuroendocrine prostate cancer
Source: Nat Commun. 2021 Mar 30;12:1979. doi: 10.1038/s41467-021-22139-7 (PMC8010057; doi:10.1038/s41467-021-22139-7)
Supplement: Supplementary file 2 — Description of Additional Supplementary Files [file 41467_2021_22139_MOESM2_ESM.pdf]

Description of Additional Supplementary Files

Supplementary Data 1: Summary statistics for ChIP-seq experiments

Supplementary Data 2: NEPC- and PRAD-enriched candidate regulatory elements

Supplementary Data 3: H3K27ac HiChIP loop calls for LNCaP

Supplementary Data 4: H3K27ac HiChIP loop calls for LuCaP 173.1
