# Supplementary material for: Reprogramming of the FOXA1 cistrome in treatment-emergent neuroendocrine prostate cancer
Source: Nat Commun. 2021 Mar 30;12:1979. doi: 10.1038/s41467-021-22139-7 (PMC8010057; doi:10.1038/s41467-021-22139-7)
Supplement: Supplementary file 4 — Reporting Summary [file 41467_2021_22139_MOESM4_ESM.pdf]

nature research

Corresponding author(s): Matthew L. Freedman

Last updated by author(s): 1-18-21

Reporting Summary

Nature Research wishes to improve the reproducibility of the work that we publish. This form provides structure for consistency and transparency in reporting. For further information on Nature Research policies, see our Editorial Policies and the Editorial Policy Checklist.

Statistics

For all statistical analyses, confirm that the following items are present in the figure legend, table legend, main text, or Methods section.

n/a	Confirmed	
☐	☒	The exact sample size (n) for each experimental group/condition, given as a discrete number and unit of measurement
☐	☒	A statement on whether measurements were taken from distinct samples or whether the same sample was measured repeatedly
☐	☒	The statistical test(s) used AND whether they are one- or two-sided<br>*Only common tests should be described solely by name; describe more complex techniques in the Methods section.*
☒	☐	A description of all covariates tested
☐	☒	A description of any assumptions or corrections, such as tests of normality and adjustment for multiple comparisons
☐	☒	A full description of the statistical parameters including central tendency (e.g. means) or other basic estimates (e.g. regression coefficient) AND variation (e.g. standard deviation) or associated estimates of uncertainty (e.g. confidence intervals)
☐	☒	For null hypothesis testing, the test statistic (e.g. F, t, r) with confidence intervals, effect sizes, degrees of freedom and P value noted<br>*Give P values as exact values whenever suitable.*
☒	☐	For Bayesian analysis, information on the choice of priors and Markov chain Monte Carlo settings
☒	☐	For hierarchical and complex designs, identification of the appropriate level for tests and full reporting of outcomes
☒	☐	Estimates of effect sizes (e.g. Cohen's d, Pearson's r), indicating how they were calculated

Our web collection on statistics for biologists contains articles on many of the points above.

Software and code

Policy information about availability of computer code

Data collection	Raw Illumina output was converted to fastq format using Illumina Bcl2fastq v2.18.
Data analysis	All software or code used in this study, along with version numbers, is described in the methods section. We used BWA 0.7.17 (http://bio-bwa.sourceforge.net/) for mapping ChIP-seq data; STAR v 2.7.0f for mapping RNAseq data; MACS2 v2.1.1.20140616 (http://liulab.dfci.harvard.edu/MACS/) for peak calling; DEseq2 1.14.1 (https://bioconductor.org/packages/release/bioc/html/DESeq2.html) and Cobra 2.0 (https://bitbucket.org/cfce/cobra/src/master/) for differential peak analysis; Homer v4.7 (http://homer.ucsd.edu/homer/motif/) for motif enrichment analysis; GREAT V3.0 (http://great.stanford.edu/public/html/index.php) for genomic regions enrichment annotations; deepTools v3.3.1 and IGV v2.8.2 for ChIP-seq and methylation data visualization; BEDTools v2.26.0 to intersect and merge genomic intervals; Bismark v0.19.069 and bsseq (https://bioconductor.org/packages/release/bioc/html/bsseq.html) for processing WGBS data.

For manuscripts utilizing custom algorithms or software that are central to the research but not yet described in published literature, software must be made available to editors and reviewers. We strongly encourage code deposition in a community repository (e.g. GitHub). See the Nature Research guidelines for submitting code & software for further information.

Data

Policy information about availability of data

All manuscripts must include a data availability statement. This statement should provide the following information, where applicable:

- Accession codes, unique identifiers, or web links for publicly available datasets
- A list of figures that have associated raw data
- A description of any restrictions on data availability

Data have been deposited in GEO under accession number GSE161948 (https://www.ncbi.nlm.nih.gov/geo/query/acc.cgi?acc=GSE161948)

Field-specific reporting

Please select the one below that is the best fit for your research. If you are not sure, read the appropriate sections before making your selection.

- [x] Life sciences
- [ ] Behavioural & social sciences
- [ ] Ecological, evolutionary & environmental sciences

For a reference copy of the document with all sections, see nature.com/documents/nr-reporting-summary-flat.pdf

Life sciences study design

All studies must disclose on these points even when the disclosure is negative.

Sample size	All LuCaP samples available were profiled to maximize sample size. Based on previous analyses (Pomerantz et al., Nature Genetics, 2015; Stelloo et al., EMBO Mol Med, 2015), this sample size is sufficient for identifying cistromic changes in human cancers.
Data exclusions	No data were excluded.
Replication	FOXA1 knock-down experiments were performed twice independently with similar results. ChIP-seq and RNA-seq from cell lines with ASCL1 results were performed across two independent experiments with similar results.
Randomization	Randomization does not apply for this study because comparisons were across distinct clinical states
Blinding	Immunohistochemistry for FOXA1/FOXA2 was performed in a blinded fashion. All unsupervised analyses described in the study, such as cistrome-wide analyses across states, were blinded as to clinical state of each specimen.

Reporting for specific materials, systems and methods

We require information from authors about some types of materials, experimental systems and methods used in many studies. Here, indicate whether each material, system or method listed is relevant to your study. If you are not sure if a list item applies to your research, read the appropriate section before selecting a response.

Materials & experimental systems

n/a	Involved in the study	
[ ]	[x]	Antibodies
[ ]	[x]	Eukaryotic cell lines
[x]	[ ]	Palaeontology and archaeology
[x]	[ ]	Animals and other organisms
[ ]	[x]	Human research participants
[x]	[ ]	Clinical data
[x]	[ ]	Dual use research of concern

Methods

n/a	Involved in the study	
[ ]	[x]	ChIP-seq
[x]	[ ]	Flow cytometry
[x]	[ ]	MRI-based neuroimaging

Antibodies

Antibodies used	The following antibodies were used: H3K27ac, Diagenode, C15410196; H3K27me3, Cell Signaling Technology, 9733S; H3K4me3, Diagenode, C15410003 premium; FOXA1, Abcam, ab23738; FOXA1, Cell Signaling Technology, 58613S; ASCL1, Abcam, ab74065; β-actin, Cell Signaling Technology, 8457L; Synaptophysin, Cell Marque, MRQ-40; INSM1, Santa Cruz, sc-377428; FOXA2, Abcam, ab108422; Chromogranin A, Abcam, ab15160; Vinculin, Cell signaling, #13901.
Validation	FOXA1 (Abcam): reference - PMID 26457646; product datasheet - https://www.abcam.com/foxa1-antibody-chip-grade-ab23738.pdf<br>FOXA1 (Cell Signaling): https://www.cellsignal.com/products/primary-antibodies/foxa1-hnf3a-d7p9b-rabbit-mab/58613<br>H3K27Ac: reference - PMID 30773341; product datasheet - https://www.diagenode.com/en/documents/datasheeth3k27ac-C15410196<br>H3K27me3: http://media.cellsignal.com/pdf/9733.pdf<br>H3K4me3: https://www.diagenode.com/en/p/h3k4me3-polyclonal-antibody-premium-50-ug-50-ul#<br>Vinclulin: https://media.cellsignal.com/pdf/13901.pdf<br>FOXA2: https://www.abcam.com/foxa2-antibody-epr4466-ab108422.html<br>ASCL1: https://www.abcam.com/mash1achaete-scute-homolog-1-antibody-ab74065.html<br>Beta-actin: https://www.cellsignal.com/products/primary-antibodies/b-actin-d6a8-rabbit-mab/8457<br>synaptophysin: https://www.sigmaaldrich.com/catalog/product/sigma/336r9?lang=en&region=US<br>INSM1: https://www.scbt.com/p/insm1-antibody-c-1<br>chromogranin: https://www.abcam.com/chromogranin-a-antibody-ab15160.html

April 2020

Eukaryotic cell lines

Policy information about cell lines

Cell line source(s)	LNCaP cells were obtained from ATCC. LNCaP 42D and 42F were derived from LNCaP from Dr. Amina Zoubeidi's lab. Available upon request from Dr. Amina Zoubeidi (not commercially available currently).
Authentication	Cell line identity for LNCaP 42D and 42F was confirmed using short tandem repeat profiling.
Mycoplasma contamination	Cell lines were not tested for mycoplasma contamination.
Commonly misidentified lines (See ICLAC register)	No commonly misidentified cell lines were used in the study

Human research participants

Policy information about studies involving human research participants

Population characteristics	Two fresh-frozen biopsies from metastatic prostate cancer from adult (≥ 18 years) males were selected from the Dana-Farber Cancer Institute (DFCI) Gelb Center biobank and database. The subjects were enrolled on DFCI Protocol 01-045, approved by the Dana-Farber Cancer Institute/Harvard Cancer Center IRB. As described in Nguyen et al, Prostate, 2017 (PMID 28156002), production of PDXs, derived from adult male prostate cancer metastases, was approved by the University of Washington Human Subjects Division IRB, which approved all Informed Consents that were used for tissue acquisition (IRB #39053).
Recruitment	LuCaPs were derived from patients (predominantly of European ancestry) who were treated for metastatic prostate cancer at the University of Washington as described in Nguyen et al, Prostate, 2017 (PMID 28156002). The two metastatic biopsy specimens profiled here were from men treated at the Dana-Farber Cancer Institute for metastatic prostate cancer who consented to tissue banking protocols (offered to all patients seen at the center).
Ethics oversight	The Dana-Farber Cancer Institute/Harvard Cancer Center IRB, University of Washington Human Subjects Division IRB,

Note that full information on the approval of the study protocol must also be provided in the manuscript.

ChIP-seq

Data deposition

- [x] Confirm that both raw and final processed data have been deposited in a public database such as GEO.
- [x] Confirm that you have deposited or provided access to graph files (e.g. BED files) for the called peaks.

Data access links *May remain private before publication.*	https://www.ncbi.nlm.nih.gov/geo/query/acc.cgi?acc=GSE161948
Files in database submission	LuCaP_136CR_H3K27ac.bed<br>LuCaP_136_H3K27ac.bed<br>LuCaP_141_H3K27ac.bed<br>LuCaP_145.1_H3K27ac.bed<br>LuCaP_145.2_H3K27ac.bed<br>LuCaP_167_H3K27ac.bed<br>LuCaP_170.2_H3K27ac.bed<br>LuCaP_170.3_H3K27ac.bed<br>LuCaP_173.1_H3K27ac.bed<br>LuCaP_173.2_H3K27ac.bed<br>LuCaP_176_H3K27ac.bed<br>LuCaP_189.3_H3K27ac.bed<br>LuCaP_189.4_H3K27ac.bed<br>LuCaP_35_H3K27ac.bed<br>LuCaP_49_H3K27ac.bed<br>LuCaP_58_H3K27ac.bed<br>LuCaP_70CR_H3K27ac.bed<br>LuCaP_70_H3K27ac.bed<br>LuCaP_77CR_H3K27ac.bed<br>LuCaP_77_H3K27ac.bed<br>LuCaP_78CR_H3K27ac.bed<br>LuCaP_78_H3K27ac.bed<br>LuCaP_81CR_H3K27ac.bed<br>LuCaP_81_H3K27ac.bed<br>LuCaP_86.2_H3K27ac.bed<br>LuCaP_92_H3K27ac.bed<br>LuCaP_93_H3K27ac.bed

LuCaP_136CR_FOXA1.bed
LuCaP_136_FOXA1.bed
LuCaP_141_FOXA1.bed
LuCaP_145.1_FOXA1.bed
LuCaP_145.2_FOXA1.bed
LuCaP_167CR_FOXA1.bed
LuCaP_167_FOXA1.bed
LuCaP_170.2_FOXA1.bed
LuCaP_170.3_FOXA1.bed
LuCaP_173.1_FOXA1.bed
LuCaP_173.2_FOXA1.bed
LuCaP_176_FOXA1.bed
LuCaP_189.3_FOXA1.bed
LuCaP_189.4_FOXA1.bed
LuCaP_208.1_FOXA1.bed
LuCaP_35_FOXA1.bed
LuCaP_49_FOXA1.bed
LuCaP_58_FOXA1.bed
LuCaP_70CR_FOXA1.bed
LuCaP_70_FOXA1.bed
LuCaP_77CR_FOXA1.bed
LuCaP_77_FOXA1.bed
LuCaP_78CR_FOXA1.bed
LuCaP_78_FOXA1.bed
LuCaP_81CR_FOXA1.bed
LuCaP_81_FOXA1.bed
LuCaP_86.2_FOXA1.bed
LuCaP_92_FOXA1.bed
LuCaP_93_FOXA1.bed
LuCaP_145.1_H3K4me3.bed
LuCaP_145.2_H3K4me3.bed
LuCaP_173.1_H3K4me3.bed
LuCaP_49_H3K4me3.bed
LuCaP_58_H3K4me3.bed
LuCaP_77_H3K4me3.bed
LuCaP_78_H3K4me3.bed
LuCaP_81_H3K4me3.bed
LuCaP_92_H3K4me3.bed
LuCaP_93_H3K4me3.bed
LuCaP_145.1_H3K27me3.bed
LuCaP_145.2_H3K27me3.bed
LuCaP_173.1_H3K27me3.bed
LuCaP_49_H3K27me3.bed
LuCaP_58_H3K27me3.bed
LuCaP_77_H3K27me3.bed
LuCaP_78_H3K27me3.bed
LuCaP_81_H3K27me3.bed
LuCaP_92_H3K27me3.bed
LuCaP_93_H3K27me3.bed
LuCaP_136CR_H3K27ac.bw
LuCaP_136_H3K27ac.bw
LuCaP_141_H3K27ac.bw
LuCaP_145.1_H3K27ac.bw
LuCaP_145.2_H3K27ac.bw
LuCaP_167_H3K27ac.bw
LuCaP_170.2_H3K27ac.bw
LuCaP_170.3_H3K27ac.bw
LuCaP_173.1_H3K27ac.bw
LuCaP_173.2_H3K27ac.bw
LuCaP_176_H3K27ac.bw
LuCaP_189.3_H3K27ac.bw
LuCaP_189.4_H3K27ac.bw
LuCaP_35_H3K27ac.bw
LuCaP_49_H3K27ac.bw
LuCaP_58_H3K27ac.bw
LuCaP_70CR_H3K27ac.bw
LuCaP_70_H3K27ac.bw
LuCaP_77CR_H3K27ac.bw

LuCaP_77_H3K27ac.bw
LuCaP_78CR_H3K27ac.bw
LuCaP_78_H3K27ac.bw
LuCaP_81CR_H3K27ac.bw
LuCaP_81_H3K27ac.bw
LuCaP_86.2_H3K27ac.bw
LuCaP_92_H3K27ac.bw
LuCaP_93_H3K27ac.bw
LuCaP_136CR_FOXA1.bw
LuCaP_136_FOXA1.bw
LuCaP_141_FOXA1.bw
LuCaP_145.1_FOXA1.bw
LuCaP_145.2_FOXA1.bw
LuCaP_167CR_FOXA1.bw
LuCaP_167_FOXA1.bw
LuCaP_170.2_FOXA1.bw
LuCaP_170.3_FOXA1.bw
LuCaP_173.1_FOXA1.bw
LuCaP_173.2_FOXA1.bw
LuCaP_176_FOXA1.bw
LuCaP_189.3_FOXA1.bw
LuCaP_189.4_FOXA1.bw
LuCaP_208.1_FOXA1.bw
LuCaP_35_FOXA1.bw
LuCaP_49_FOXA1.bw
LuCaP_58_FOXA1.bw
LuCaP_70CR_FOXA1.bw
LuCaP_70_FOXA1.bw
LuCaP_77CR_FOXA1.bw
LuCaP_77_FOXA1.bw
LuCaP_78CR_FOXA1.bw
LuCaP_78_FOXA1.bw
LuCaP_81CR_FOXA1.bw
LuCaP_81_FOXA1.bw
LuCaP_86.2_FOXA1.bw
LuCaP_92_FOXA1.bw
LuCaP_93_FOXA1.bw
LuCaP_145.1_H3K4me3.bw
LuCaP_145.2_H3K4me3.bw
LuCaP_173.1_H3K4me3.bw
LuCaP_49_H3K4me3.bw
LuCaP_58_H3K4me3.bw
LuCaP_77_H3K4me3.bw
LuCaP_78_H3K4me3.bw
LuCaP_81_H3K4me3.bw
LuCaP_92_H3K4me3.bw
LuCaP_93_H3K4me3.bw
LuCaP_145.1_H3K27me3.bw
LuCaP_145.2_H3K27me3.bw
LuCaP_173.1_H3K27me3.bw
LuCaP_49_H3K27me3.bw
LuCaP_58_H3K27me3.bw
LuCaP_77_H3K27me3.bw
LuCaP_78_H3K27me3.bw
LuCaP_81_H3K27me3.bw
LuCaP_92_H3K27me3.bw
LuCaP_93_H3K27me3.bw

Genome browser session (e.g. UCSC)

No longer applicable.

Methodology

Replicates

ChIP-seq experiments in LNCaP with overexpression of transcription factors was performed in duplicate.

Sequencing depth

150bp paired-end sequencing was performed on the Illumina platform. An average of 80.1 million reads were mapped per LuCaP ChIP-seq dataset.

Antibodies	The following antibodies were used: H3K27ac, Diagenode, C15410196; H3K27me3, Cell Signaling Technology, 9733S; H3K4me3, Diagenode, C15410003 premium; FOXA1, Abcam, ab23738; FOXA1, Cell Signaling Technology, 58613S; ASCL1, Abcam, ab74065
Peak calling parameters	Narrow peaks were called on deduplicated bam files using the following command: macs2 callpeak --SPMR -B -q 0.01 --keep-dup 1 -g hs -f BAMPE --extsize 146 --nomodel -t {treat.bam} -c {input.bam}. Broad peak calls for H3K27me3 were called using: macs2 callpeak -t {treat.bam} -c {input.bam} --broad
Data quality	ChIP-seq data were shown to be of high quality by multiple measures, including peak number, fraction of reads in peaks (FRiP score), number of peaks with >10-fold or >20-fold enrichment. narrowPeak calls contained an average of 21,684 peaks with >10-fold enrichment. Metrics for individuals samples are listed in Table S1.
Software	MACS v2.1.1.20160309
